# Divergent AKT Signalling Mechanisms Regulate GLUT4 Translocation and Glucose Uptake in Skeletal Muscle and Adipose Tissue

**DOI:** 10.1002/jcsm.70335

**Published:** 2026-07-08

**Authors:** Natasha Jaiswal, Matthew Gavin, Louise Lantier, Olivia Yu Yu Ong, David H. Wasserman, Paul M. Titchenell

**Affiliations:** ^1^ Institute for Diabetes, Obesity, and Metabolism Perelman School of Medicine at the University of Pennsylvania Philadelphia Pennsylvania USA; ^2^ Department of Physiology Perelman School of Medicine at the University of Pennsylvania Philadelphia Pennsylvania USA; ^3^ Department of Health and Kinesiology Purdue University West Lafayette USA; ^4^ Department of Molecular Physiology and Biophysics Vanderbilt University School of Medicine Nashville Tennessee USA; ^5^ Vanderbilt Mouse Metabolic Phenotyping Center Vanderbilt University School of Medicine Nashville Tennessee USA

**Keywords:** AKT signalling, AMPK signalling, GLUT4 translocation, insulin signalling, PI3K‐PIP3 pathway

## Abstract

**Background:**

The serine/threonine kinase AKT is a key regulator of glucose and energy metabolism. Prevailing dogma suggests that AKT is an obligate intermediate for glucose uptake in all metabolic tissues and that impaired AKT signalling is a major molecular driver of insulin resistance in obesity. However, whether AKT is universally required for insulin‐stimulated glucose uptake across tissues in vivo has remained unresolved.

**Method:**

Several mouse models of adipose‐specific AKT2 deletion (F‐AKT2KO) and skeletal muscle‐specific AKT1, AKT2 and combined AKT1/AKT2 knockout mice (M‐AKT1KO, M‐AKT2KO and M‐AKTDKO) were generated. Skeletal muscle and adipose tissues were analysed following in vivo administration of insulin (2 U/kg), using Western blotting, phosphoproteomics, PI(3,4,5)P3 ELISA and mitochondrial respiration assays. Glucose metabolism was assessed using [^3^H]‐2‐deoxyglucose uptake, hyperinsulinemic‐euglycemic clamps, glucose and insulin tolerance tests. Global phosphoproteomics was performed in insulin‐stimulated skeletal muscle lacking AKT isoforms.

**Results:**

Loss of AKT2 in adipose tissue impaired insulin signalling, including reduced pAS160^Thr649^, and markedly decreased insulin‐stimulated glucose uptake (~2–3 fold reduction in F‐AKT2KO vs F‐Control, *p* < 0.001, *n* = 7–11), resulting in systemic insulin resistance. In contrast, M‐AKTDKO mice exhibited a robust increase in insulin‐stimulated glucose uptake (~3–4 fold increase) despite complete loss of AKT signalling, including pAS160^Thr649^. Phosphoproteomic analysis of M‐AKTDKO (*n* = 3–4) identified ~7088 phosphosites, with 795 uniquely upregulated in insulin‐stimulated M‐AKTDKO muscle (fold change > 2, *p* < 0.05), enriched in PI3K and AMPK pathways. Consistently, ~8‐fold (*p* < 0.05) increase in PIP3 levels was observed in M‐AKTDKO muscle in response to insulin. Additionally, AKT deficiency was associated with reduced complex I–dependent mitochondrial respiration (~37% decrease in state 3 respiration), consistent with altered energetic status and AMPK activation. Genetic epistasis experiments demonstrated that both AKT and AMPK activity are required for insulin‐stimulated glucose uptake, systemic glucose homeostasis and whole body insulin sensitivity.

**Conclusion:**

These findings challenge the long‐standing assumption that AKT is universally required for insulin‐stimulated glucose uptake in vivo. The study demonstrates that while AKT is essential in adipose tissue, it is dispensable for insulin‐stimulated glucose uptake in skeletal muscle. AKT exerts negative feedback on PI3K signalling in both tissues; however, only skeletal muscle engages AMPK in the abscence of AKT to preserve glucose uptake. These findings redefine tissue‐specific insulin signalling mechanisms and identify AMPK as a critical downstream target of PI3K that coordinates with AKT to regulate glucose uptake.

## 1 Introduction

Insulin maintains systemic glucose homeostasis by suppressing hepatic glucose production and promoting glucose uptake in skeletal muscle and adipose tissue. Insulin resistance, characterised by impaired glucose regulation, is a key feature of metabolic diseases such as type 2 diabetes. In humans, failure of insulin‐stimulated skeletal muscle glucose uptake is considered a primary cause of insulin resistance. Therefore, there is considerable interest in understanding the underlying mechanisms mediating insulin's control of skeletal muscle glucose metabolism.

At the molecular level, insulin binding to the insulin receptor induces receptor autophosphorylation and recruitment of IRS1/IRS2 via their phosphotyrosine‐binding domains. Tyrosine‐phosphorylated IRS proteins then activate phosphoinositide 3‐kinase (PI3K) to generate phosphatidylinositol (3,4,5)‐trisphosphate (PIP3) at the plasma membrane. PIP3 recruits PDK1 and the serine/threonine kinase AKT, enabling their activation. AKT exists in three isoforms in mammals: AKT1, AKT2 and AKT3 (also known as PKB a, β, γ), each encoded by a distinct gene. AKT1 is ubiquitously expressed, AKT2 is enriched in metabolic tissues such as skeletal muscle, adipose tissue and liver, and AKT3 is expressed in brain and testis. Although whole‐body AKT1 or AKT3 deletion produces growth phenotypes without major metabolic impairment, AKT2 deficiency results in systemic insulin resistance, impaired glucose tolerance and reduced muscle glucose uptake [1, 2, 3, 4]. Notably, rare dominant‐negative mutations in *Akt2* are associated with severe diabetes in humans [5], whereas mosaic activating AKT2 mutations induce recurrent hypoglycaemia and asymmetric overgrowth [6]. Consistent with these genetic studies, dysregulated AKT activity is observed in individuals with insulin resistance and type 2 diabetes [7, 8, 9]. Thus, AKT2 is suggested to be the major isoform required for metabolic insulin action [10].

Seminal in vitro studies in cultured adipocytes demonstrated that AKT activity is required for insulin‐stimulated glucose uptake, and pharmacological AKT inhibition suppresses insulin‐dependent glucose transport in skeletal muscle ex vivo [11, 12, 13]. Thus, a common hypothesis prevails in the field that AKT‐mediated signalling is an obligate requirement for insulin‐stimulated glucose transport and utilisation across all metabolic tissues, including skeletal muscle and adipose tissue. Mechanistically, in vitro studies in adipocytes suggest that AKT phosphorylates the Rab‐GAP TBC1D4 (AS160) and its paralog TBC1D1. This promotes 14–3‐3 binding, relieves their inhibitory constraints on GLUT4 vesicle trafficking and enables GLUT4 translocation. Consistent with this mechanism, loss‐of‐function mutations in human TBC1D4 cause insulin resistance [9, 14]. Given that common AKT substrates are expressed in both rodent and human skeletal muscle and adipose tissue, AKT is widely assumed to regulate a shared downstream mechanism governing insulin‐stimulated glucose uptake. However, emerging studies in both mice and humans suggest that insulin can regulate glucose transport in skeletal muscle independent of the AKT‐AS160 pathway [15, 16, 17, 18]. Thus, the role of AKT as the central regulator of metabolic actions of insulin in skeletal muscle and adipose tissue remains controversial.

Here, we investigate tissue‐specific roles of AKT isoforms in skeletal muscle and adipose tissue in vivo. We confirm that AKT2 is an obligate intermediate involved in adipocyte insulin action and glucose uptake. Interestingly, we find that AKT is largely dispensable for insulin‐stimulated glucose uptake in skeletal muscle, and we uncover a previously unrecognised role of AKT in limiting PI3K–PIP3 signalling and controlling downstream AMPK activation. Using genetic epistasis experiments, we demonstrate that both AKT and AMPK are required for insulin‐stimulated glucose uptake in muscle. These findings reveal a tissue‐specific division of labour between AKT and AMPK and challenge the longstanding view that AKT is the central and universal mediator of insulin action in all metabolic tissues.

## 2 Materials and Methods

### 2.1 Mice

Skeletal muscle‐specific congenital and inducible knockout mice were generated using Cre‐LoxP recombination approach, as described earlier [17]. Briefly, the floxed strains *Akt1*_*loxP/loxP*_, *Akt2*_*loxP/loxP*_, *Akt1*_*loxP/loxP*_ *;Akt2*_*loxP/loxP*_, *AMPK* a *1*_*loxP/loxP*_ *, AMPK* a *2*_*loxP/loxP*_, or *Akt1*_*loxP/loxP*_ *;* *Akt2_loxP/loxP_;* *AMPK* a *1*_*loxP/loxP*_ *;* *AMPK* a *2*_*loxP/loxP*_ were crossed with mice carrying the Cre recombinase under the control of the skeletal muscle actin promoter, ACTA1‐Cre (Jackson Laboratory, stock number 006149) to generate skeletal muscle‐specific knockout mice. Littermate lacking the Cre transgene served as controls [17, 19]. Twelve‐week‐old male mice were used for all experiments. For skeletal muscle AKT deficiency in adult mice, floxed mice were crossed to mice containing the Cre recombinase‐estrogen receptor fusion protein under the control of human ACTA‐1 (HSA‐ESR‐CRE) (Jackson Laboratory, stock number 031934), allowing tamoxifen‐inducible Cre‐mediated recombination. Floxed AKT/AMPK mice (12 weeks of age) lacking the HSA‐ESR‐CRE transgene (M‐indControl) or carrying the transgene (M‐indAKTDKO/M‐AMPK) were injected with tamoxifen (100 mg/kg, intraperitoneally) once daily for 5 consecutive days to induce knockout. All experiments were performed 4 weeks later following the 5th day of tamoxifen. Adipose‐specific AKT2 knockout mice (F‐AKT2KO) were generated by crossing Adipoq *Cre* positive male mice heterozygous for *Akt2*_*loxP/loxP*_ to females *Akt2*_*loxP/loxP*_ [20]. Littermates lacking Cre transgene served as controls. All the experiments were performed in 12‐week‐old mice unless otherwise indicated. All animal studies were reviewed and approved by the University of Pennsylvania Institutional Animal Care and Use Committee (IACUC) and were conducted in accordance with National Institute of Health (NIH) guidelines for the care and use of laboratory animals.

### 2.2 Ex Vivo Glucose Uptake

Glucose uptake was measured in EDL and soleus strips using [^3^H]‐2‐deoxyglucose, as previously described. White pre‐adipocytes were isolated as described previously [21] and insulin stimulated glucose uptake in primary adipocytes was determined using [^14^C]‐D‐glucose. All ex vivo experiments were performed on muscles from mice fasted for 4–6 h before tissue harvest to minimise baseline variability in glucose metabolism.

### 2.3 PI(3,4,5)P3 and PI(4,5)P2 Mass ELISA

M‐Control and M‐AKTDKO gastrocnemius muscles were harvested from mice fasted overnight and then stimulated with insulin (2 U/kg body weight) for 10 or 20 min. Lipids were extracted and measured using an ELISA based assay (Echelon Biosciences) as per the manufacturer's instructions [22, 23] and normalised to the muscle weight.

### 2.4 Hyperinsulinemic‐Euglycemic (Insulin) Clamp

Hyperinsulinemic‐euglycemic clamps were performed in catheterised mice at the Vanderbilt University school of medicine as previously described [24]. Human insulin was infused at the rate of 2.5 mU/Kg/min and blood glucose was maintained between 120 mg/dL and 140 mg/dL by infusing 20% glucose at various rates. Whole‐body glucose turnover and tissue‐specific uptake were measured using [^3^H]2‐deoxyglucose infusion. Plasma and tissue were processed as described previously [25]. Skeletal muscle glucose storage was calculated as the difference between Rd. and glycolytic rate [26].

### 2.5 GLUT4 Lentivirus Preparation and Ex Vivo GLUT4 Translocation Assay

Lenti‐myc‐GLUT4‐mCherry lentivirus was packaged with a third‐generation lentivirus system, as previously described [27, 28]. GLUT4 translocation to the cell surface in single muscle fibres isolated from the flexor digitorum brevis (FDB) muscle was measured as described previously [28]. Briefly, single FDB fibres from Control and knockout mice were transduced with lenti‐myc‐GLUT4‐mCherry lentivirus, incubated with or without insulin (100 nM) for 15 min. Non‐permeabilised FDB fibres were then fixed in 4% PFA for 10 min, stained with Myc‐Tag (71D10) rabbit monoclonal antibody (Cell Signalling Technology, 2278) overnight (1:500), followed by anti‐rabbit secondary antibody, Alex 488 (Invitrogen, A11034) for 1 h (1:1000) to measure the abundance of GLUT4 expressed on the plasma membrane. Red (mCherry) indicates total expression of the lenti‐myc‐GLUT4‐mCherry lentivirus. Images were collected using Keyence BZ‐X700 fluorescent light microscope with 20x objective.

### 2.6 Mitochondrial Respiration

Mitochondrial respiration rates were measured in mitochondria isolated from the gastrocnemius muscles of M‐Control and M‐AKTDKO mice using an Oroboros Oxygraph‐2k high‐resolution respirometer, as described earlier [17].

### 2.7 C2C12 Cell Culture

C2C12 myoblasts at ~70%–80% confluency were differentiated into myotubes using differentiation medium (DMEM with 2% horse serum). Post differentiation for 5–6 days, myotubes were serum and glucose deprived for 24 h. The cells were treated with 10 μM MK2206 for 6 h followed by 10 nM insulin stimulation for 15 min and harvested in 1× RIPA buffer.

### 2.8 Statistics

All data are presented as mean ± SEM. Statistical analysis was performed using one‐way ANOVA when more than two groups were compared, two‐way ANOVA when two conditions were involved, and unpaired, two‐tailed Student's *t* test when only two groups of data were concerned. *p* ≤ 0.05 was considered statistically significant.

## 3 Results

### 3.1 Differential Effects of AKT2 Signalling on Muscle and Adipose Insulin‐Sensitivity In Vivo

**FIGURE 1:**
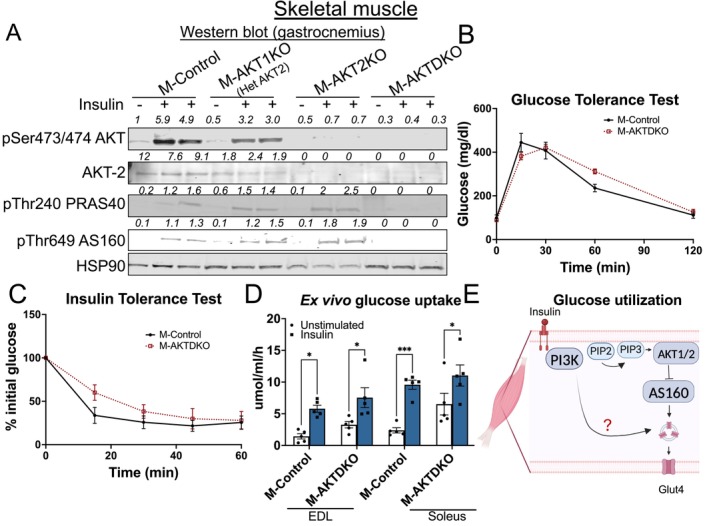
Canonical AKT2‐AS160 is dispensable for glucose uptake in skeletal muscle. (a) Western blot for phosphorylation status of AKT1, AKT2, PRAS40 and AS160 in gastrocnemius muscle from M‐Control and knockout mice either unstimulated or stimulated with insulin (2 U/kg) for 20 min following an overnight fast. Densitometric analysis of band intensities was performed using ImageJ, normalised to HSP90 as a loading control and expressed as fold change relative to unstimulated control; values are shown above each western blot. (b) Intraperitoneal glucose tolerance test (2 g/kg) (*n* = 4 for control and M‐AKTDKO mice). (c) Insulin tolerance test (0.75 U/kg) (*n* = 4). (d) Ex vivo insulin‐stimulated glucose uptake was measured in EDL or soleus from M‐Control and M‐AKTDKO mice (*n* = 5). (e) Hypothesised model of insulin‐stimulated glucose uptake via PI3K‐mediated mechanism in skeletal muscle (*p < 0.05, ***p < 0.001 vs. M‐Control).

To determine the relative impact of AKT deficiency on downstream insulin signalling, insulin (2 U/kg body weight) was administered to M‐Control, M‐AKT1KO (heterozygous for AKT2), M‐AKT2KO, M‐AKTDKO and F‐AKT2KO mice following 16 h fasting. Gastrocnemius muscles or epididymal white adipose tissue (eWAT) were harvested 20 min later and subjected to western blot analysis. Muscle‐specific deletion of AKT1 alone, which accounts for less than 10% of the AKT expressed in skeletal muscle, did not affect insulin signalling as canonical targets of AKT such as pPRAS40^Thr240^ and pAS160^Thr649^ were not different from control muscle (Figure 1A). Notably, deletion of AKT1 alone has previously been reported not to affect growth or glucose homeostasis [4, 29, 30]. Surprisingly, M‐AKT2KO muscle also exhibited similar phosphorylation of pPRAS40^Thr240^ and pAS160^Thr649^ in response to a bolus of insulin, despite a dramatic reduction in pAKT^Ser474^ determined using an AKT2‐phospho specific antibody. This is consistent with our previous findings that indicate residual AKT1 is sufficient to preserve signalling downstream of AKT in the absence of AKT2 (Figure 1A). In line with this, insulin failed to induce pPRAS40^Thr240^ and pAS160^Thr649^ in M‐AKTDKO muscles (Figure 1A). It is noteworthy that insulin receptor in M‐AKTDKO mice demonstrated normal activation of the receptor, as reported previously [17]. These findings suggest that AKT1 functionally compensates in the absence of AKT2, and minimal amounts of phosphorylated AKT are necessary to activate downstream signalling cascades in skeletal muscle.

Our previous work demonstrated that consistent with the normal insulin signalling, M‐AKT1KO and M‐AKT2KO mice also exhibited normal glucose homeostasis as determined by glucose tolerance test, insulin tolerance test and ex vivo glucose uptake capacity in response to insulin [17]. Consistent with our previous findings, despite a complete defect in insulin signalling pathways downstream of AKT, M‐AKTDKO mice exhibit normal insulin tolerance and ex vivo glucose uptake capacity in M‐AKTDKO EDL and soleus muscles following insulin stimulation (Figure 1B–D). This was further supported by normal insulin‐mediated GLUT4 translocation in single fibres isolated from the flexor digitorum brevis (FDB) muscle in both M‐Control and M‐AKTDKO mice (Supplementary Figures 1A,B). Previously, similar phenotypes in inducible, skeletal muscle‐specific knockouts of AKT [17] have been reported, suggesting that the observed results are not attributable to any developmental or compensatory effects. In view of these observations, we hypothesise that AKT is not the sole obligate intermediate of skeletal muscle insulin signalling to regulate glucose uptake. Moreover, we postulate the existence of a parallel pathway, in addition to AKT, that regulates glucose uptake in skeletal muscle **(**Figure 1E **)**.

**FIGURE 2:**
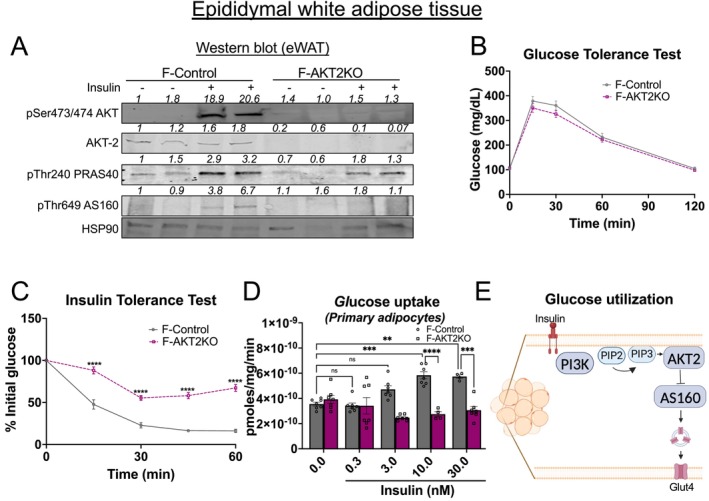
AKT2 mediated AS160 signalling is critical for glucose uptake in adipose tissue. (a) Western Blot for phosphorylation status of AKT1, AKT2, PRAS40 and AS160 in epididymal white adipose tissue (eWAT) from F‐Control and F‐AKT2KO mice either unstimulated or stimulated with insulin (2 U/kg) for 20 min following an overnight fast. Densitometric analysis of band intensities was performed, normalised to HSP90 and expressed as fold change relative to unstimulated control; values are shown above each western blot. (b) Intraperitoneal glucose tolerance test (2 g/kg) (*n* = 4 for F‐Control and F‐AKT2KO mice). (c) Insulin tolerance test (0.75 U/kg) (*n* = 4). (d) Ex vivo insulin‐stimulated glucose uptake was measured in primary adipocytes from F‐Control and F‐AKT2KO mice (*n* = 6–7). (e) Schematic of insulin‐stimulated glucose uptake in adipose tissue (**p < 0.01, ***p < 0.001 and ****p < 0.0001 vs. F‐Control).

In contrast to the skeletal muscle AKT2 knockouts, F‐AKT2KO eWATs exhibit impaired insulin signalling downstream of AKT, including reduced phosphorylation of pPRAS40^Thr240^ and pAS160^Thr649^ (Figure 2A). Of note, mice with combined deletion of both AKT1 and AKT2 (F‐AKTKO) were severely lipodystrophic at 8–12 weeks of age such that all subcutaneous and cutaneous fat depots were absent [31]. Interestingly, F‐AKT2KOs display normal glucose tolerance but impaired insulin tolerance (Figure 2B,C). Strikingly, insulin completely failed to stimulate glucose uptake in isolated primary adipocytes from F‐AKT2KO eWAT in a dose‐dependent manner (Figure 2D). These studies suggest that the AKT2 pathway is an obligate intermediate for insulin action and glucose uptake in adipocytes (Figure 2E). Altogether, our data suggest distinct biology of AKT1 and AKT2 in skeletal muscle vs. adipose tissue. Moreover, the study underscores the complexity and adaptability of insulin signalling in skeletal muscle.

### 3.2 Muscle AKT Activity Is Critical for Maintaining the Basal Phosphoproteome and Limiting AMPK Activation

**FIGURE 3:**
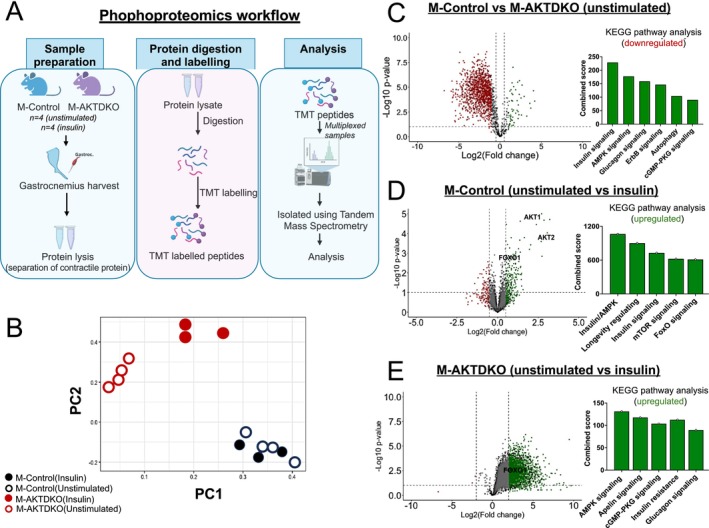
Phosphoproteome signature of AKT dependent/independent signalling in the basal and insulin‐stimulated condition. (a) Schematic representation of the workflow for phosphoproteomics sample preparation and mass spectrometry from gastrocnemius muscle. (b) Principal component analysis (PCA) for phosphoproteomics samples showing the effect of both M‐AKTDKO and insulin on skeletal muscle phosphorylation levels (*n* = 3–4). (c–e) Volcano plots of log_2_ (fold change) and −log10 *p* value (cutoff > 1 or *p* value 0.05) in M‐Control and M‐AKTDKO muscles in ± insulin‐stimulated condition. Red dots represent significantly downregulated phosphopeptides, green dots represent significantly upregulated phosphopeptides (*p* < 0.05, *n* = 4, *t*‐test). Grey dots represent phosphopeptides that were detected but not significantly altered. Enrichr‐based enrichment analysis using the genes corresponding to the significant phosphosites differentially expressed. (c) Phosphopeptide changes between M‐Control and M‐AKTDKO in unstimulated (basal) condition. Enrichr‐based top six enriched pathways according to KEGG database plotted against the combined score (input is the genes corresponding to significantly downregulated phosphopeptides; cutoff: Log_2_(fold‐change) > 1 or *p* value ≤ 0.05). (d) Volcano plot demonstrating Log_2_(fold‐change) difference in phosphopeptides plotted against the −log10 *p* value in M‐Control muscle in response to insulin stimulation (20 min) compared to unstimulated condition. Top five enriched pathways according to KEGG database were ranked by the combined score using Enrichr (input is the genes corresponding to significantly upregulated phosphopeptides; cutoff: Log_2_(fold‐change) > 1 or *p* value 0.05). (e) Volcano plot demonstrating Log_2_(fold‐change) difference in phosphopeptides plotted against the −log10 *p* value in M‐AKTDKO muscle in response to insulin stimulation (20 min) compared to unstimulated muscle. Top five enriched pathways according to KEGG database were ranked by the combined score using Enrichr (input is the genes corresponding to significantly upregulated phosphopeptides; cutoff: Log_2_(fold‐change) > 1 or *p* value ≤ 0.05).

To identify unique AKT‐dependent/independent signalling events that might be required for the regulation of glucose homeostasis by insulin, we performed an unbiased phosphoproteomics using iTRAQ labeling. Muscles from M‐Control and M‐AKTDKO mice were collected immediately after injection with saline or insulin (2 U/kg body weight, 20 min), to broadly characterise the insulin‐regulated phosphoproteome and determine which phosphorylation events require AKT. Proteomic analysis of skeletal muscle is inherently challenging due to the presence of highly abundant contractile proteins hindering the detection of low abundant proteins [32]. To overcome this, we depleted contractile proteins by lysing the muscles with cold 1% NP40 lysis buffer and performing low‐speed centrifugation (Figure 3A). Stable isotope labelling with 16‐plex Tandem Mass Tag (TMT), phosphopeptide enrichment and phosphopeptide‐level fractionation was performed prior to tandem mass spectrometry (Figure 3A). This approach allowed a comprehensive phosphoproteome analysis in M‐Control and M‐AKTDKO muscle in unstimulated and insulin‐stimulated conditions. In total, 7088 phosphosites were identified. Principal‐component analysis (PCA) of our phosphoproteomics data was performed to obtain a global view of phosphopeptide changes with insulin treatment (Figure 3B). PCA revealed segregation of the M‐Control and M‐AKTDKO samples as well as clustering of unstimulated and insulin‐stimulated M‐AKTDKO samples, suggesting significant changes in the phosphoproteome in basal and insulin‐stimulated conditions in M‐AKTDKO muscles (Figure 3B).

To determine the role of AKT in the regulation of phosphorylation events in skeletal muscle, phosphoproteome analysis of the M‐AKTDKO and M‐Control muscles was conducted under unstimulated as well as insulin stimulated condition. Volcano plot compared Log2(fold change) of M‐AKTDKO/M‐Control with statistical significance −log10(*p* value) and shaded by the threshold of *p* value < 0.05 and fold change less than −2 or > 2. Raw values of less than 50 were omitted from the analysis. Surprisingly, phosphoproteomic analysis under basal condition revealed that among the total of 7088 identified phosphosites, 1420 differentially expressed phosphosites were observed in M‐AKTDKO vs. M‐Control (21 upregulated and 1399 downregulated phosphosites), suggesting a basal, housekeeping function for AKT signalling (Figure 3C). Pathway enrichment analysis in KEGG database on the genes of 1399 effector phosphoproteins that were downregulated in M‐AKTDKO muscles were significantly involved in metabolic pathways including insulin signalling (*p* value = 1.23E−18), AMP‐activated protein kinase (AMPK) pathway (*p* value = 1.35E−15), glucagon signalling (*p* value = 1.37E−13), EGFR1 signalling pathway (*p* value = 4.56E−19). Next, phosphosite changes in response to insulin stimulation compared to unstimulated muscle were analysed in M‐Control and M‐AKTDKO muscles (Figure 3D,E and Supplementary Figure 2A,B). As expected, KEGG pathway analysis revealed enrichment of insulin signalling pathway in M‐Control muscle in response to insulin compared to unstimulated muscle. This included activation of key molecules of insulin signalling proteins such as pAKT2^Ser474^, pAKT1^Ser473^ and pFOXO1^Ser253^ (volcano plot, Figure 3D). Interestingly, despite the complete absence of AKT mediated insulin signalling, ~795 phosphosites were uniquely upregulated in M‐AKTDKO muscle following insulin stimulation (volcano plot, Figure 3E). KEGG pathway analysis of the genes corresponding to the upregulated phosphosites revealed enrichment of metabolic pathways including AMPK, cGMP‐PKG signalling, glucagon signalling. Notably, while the canonical AKT mediated insulin signalling nodes such as pFOXO1^Ser253^ were not induced in the absence of AKT, KEGG pathway analysis still indicated enrichment of the insulin signalling pathway. These findings suggest that AKT functions to restrain or fine‐tune insulin signalling, preventing excessive or alternative pathway activation.

Collectively, these results implicate an unappreciated role of skeletal muscle AKT in the maintenance of insulin signalling in skeletal muscle both under basal and insulin‐stimulated conditions.

### 3.3 AKT Negatively Regulates PI3K Activity to Limit Downstream Propagation of Insulin Signalling

**FIGURE 4:**
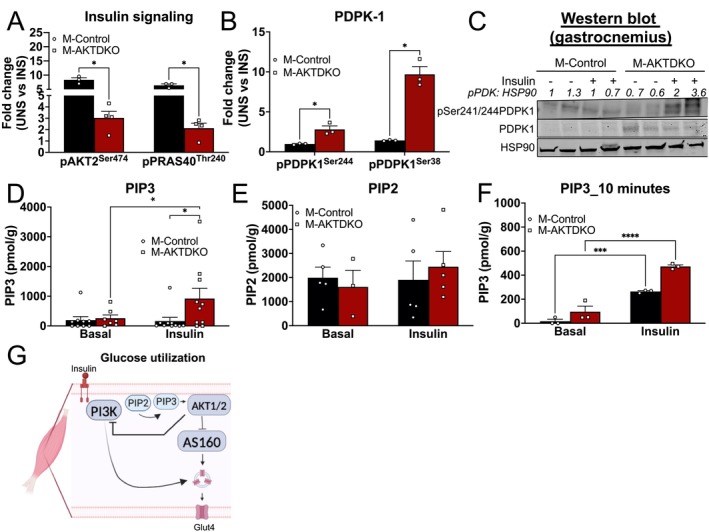
AKT negatively regulates PI3K activity to limit downstream propagation of insulin signals and maintain metabolic homeostasis. (a,b) Evaluation of the phosphopeptide changes using phosphoproteomics analysis. Graph represents the fold change of (a) the key molecules of insulin signalling pathway and (b) PDK‐1 in response to insulin (INS) stimulation compared to the unstimulated (UNS) M‐Control and M‐AKTDKO muscle. (c) Validation western Blot for phosphorylation status of PDK‐1 in M‐Control and M‐AKTDKO muscle either unstimulated or stimulated with insulin (2 U/kg) for 20 min following an overnight fast. Densitometric analysis of band intensities was performed, normalised to HSP90 and expressed as fold change relative to unstimulated control; values are shown above each western blot. (d–f) Quantification of PI(3,4,5)P3 (PIP3) and PI(3,4,5)P2 (PIP2) mass. M‐Control and M‐AKTDKO mice were treated with either BSA (basal) or insulin (2 U/kg) for 20 min or 10 min and gastrocnemius muscles were harvested. Lipids were extracted from gastrocnemius muscles and (d) PIP3 and (e) PIP2 content was measured using a ELISA based assay (*n* = 4–9). (f) PIP3 levels in gastrocnemius muscle following acute insulin stimulation (2 U/kg) for 10 (*n* = 3–4). (g) Model prediction of the insulin mediated glucose uptake in skeletal muscle that is negatively regulated by AKT (*p < 0.05, ***p < 0.001, ****p < 0.0001 vs M‐Control).

Previous studies demonstrated an essential role of PI3K in regulating muscle glucose uptake and insulin sensitivity [33]. Consistent with this, we demonstrated that the inhibition of PI3K activity in M‐AKTDKO muscles blocks insulin‐stimulated glucose uptake [17], suggesting the role of PI3K‐dependent and AKT‐independent pathway in insulin‐mediated glucose uptake in skeletal muscle. In support of this hypothesis, while the fold induction (insulin‐stimulated vs. unstimulated) of several canonical insulin signalling nodes was significantly downregulated in M‐AKTDKO muscles compared to the M‐Control muscles (Figure 4A), a significant increase in the fold change of PIP3‐dependent pPDK1^Ser38^ and pPDK1^Ser244^ in M‐AKTDKO vs. M‐Control muscles was observed (Figure 4B). Based on these findings, we hypothesise that AKT exerts negative feedback on PI3K to restrain insulin signalling and maintain glucose homeostasis. Consistent with this, western blot analysis confirmed a robust increase in pPDK1^Ser244^ expression in M‐AKTDKO muscles upon insulin stimulation (Figure 4C). To further explore this mechanism, PIP2 and PIP3 content was quantified using ELISA‐based assay as a measure of PI3K activity. M‐AKTDKO muscles exhibited a significant increase in PIP3 content in response to insulin, without affecting PIP2 content (Figure 4D,E), implicating an enhanced PI3K activity in M‐AKTDKO following AKT loss. This is consistent with the recent studies in adipocytes demonstrating an AKT‐dependent, mTORC1‐independent feedback mechanism that limits PI3K‐mediated PIP3 synthesis [34].

Interestingly, 20 min post‐insulin stimulation, PIP3 levels in control muscle were not significantly different from basal conditions (Figure 4D), despite robust downstream signalling (Figure 1A). Given the rapid turnover of PIP3, we performed a temporal analysis in vivo. At 10 min post‐insulin stimulation, a significant increase in PIP3 levels was observed in both M‐Control and M‐AKTDKO muscle, with a greater magnitude of induction in M‐AKTDKO muscle (Figure 4E). These results indicate that differences in PI3K activity are more pronounced at early time points following insulin stimulation and loss of AKT sustains PI3K activity.

Collectively, these findings indicate that AKT limits PI3K‐mediated PIP3 production through a negative feedback mechanism. Loss of AKT disrupts this feedback, resulting in sustained PIP3 activity and enhanced PIP3 accumulation (Figure 4F). Thus, we identify a previously unrecognised housekeeping role for AKT in restraining proximal insulin signalling via PI3K and maintaining basal signalling homeostasis.

### 3.4 AKT Inhibits Insulin Mediated Activation of AMPK Pathway and Promotes Mitochondrial Respiration

**FIGURE 5:**
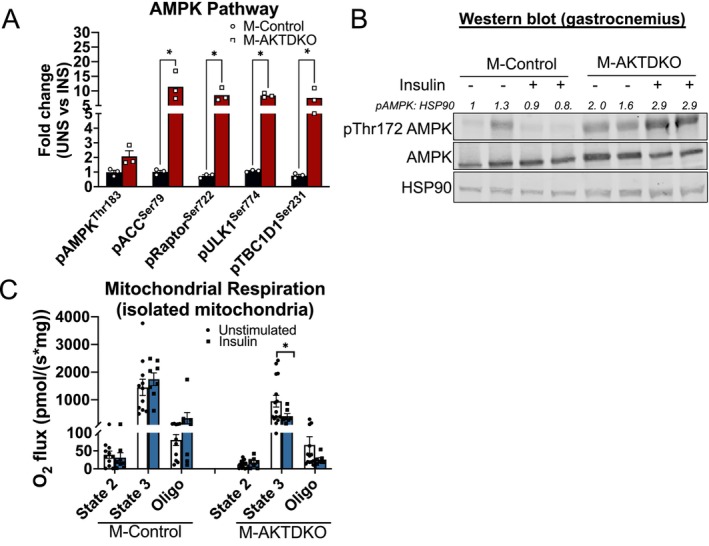
AKT prevents insulin mediated activation of AMPK pathway to maintain complex I dependent energy homeostasis. (a) Evaluation of the phosphorylation status of the key molecules of the AMPK signalling pathway during phosphoproteomic analysis. Graph represents the fold change of the key molecules of AMPK signalling in response to insulin (INS) stimulation relative to the unstimulated (UNS) M‐Control and M‐AKTDKO muscle. (b) Western blot validation of phosphoproteomics. Phosphorylation of AMPK in gastrocnemius muscle from M‐Control and M‐AKTDKO mice either unstimulated or stimulated with insulin (2 U/kg) for 20 min following an overnight fast. Densitometric analysis of band intensities was performed, normalised to HSP90, and expressed as fold change relative to unstimulated control; values are shown above each western blot. (c) Oroborus high‐capacity respirometer analysis of isolated mitochondria from M‐Control and M‐AKTDKO gastrocnemius muscle either unstimulated or stimulated with 2 U/kg insulin for 20 min (*n* = 7–10) (*p < 0.05 vs. M‐Control).

As demonstrated in Figure 3E, KEGG pathway analysis of phosphosites uniquely upregulated in insulin‐stimulated M‐AKTDKO muscle demonstrates enrichment of the AMPK pathway. Further analysis of the phosphoproteomics data in M‐AKTDKO vs. M‐Control muscle indicated the significant upregulation of the key molecules of the AMPK signalling pathway, including pACC^Ser79^, pRaptor^Ser722^, pULK1^Ser774^ and pTBC1D1^Ser231^, following insulin stimulation (Figure 5A). To validate these phosphoproteomics findings, the protein expression level of pAMPK^Thr172^ was measured using western blotting, which demonstrated a robust increase in pAMPK^Thr172^ signal in M‐AKTDKO muscle, which was further augmented upon insulin stimulation (Figure 5B).

AMPK is a well‐established regulator of glucose transport and utilisation in the contracting muscle [35, 36, 37, 38]. In vitro studies have reported direct regulation of insulin‐mediated AMPK activity via AKT‐dependent phosphorylation of the inhibitory Ser485/491 site in a variety of tissues including cardiomyocytes (heart), adipocytes, vascular smooth muscle cells and skeletal muscle [39, 40]. Consistent with these reports, enhanced phosphorylation of pAMPK^Ser485^ in response to insulin was observed in C2C12 cells, and this effect was abolished following treatment with MK2206, a specific AKT inhibitor (Supplementary Figure 3A). Despite these cellular findings, no difference in phosphorylation of pAMPK^Ser485^ was detected in gastrocnemius muscles of M‐Control or M‐AKTDKO in response to insulin stimulation (Supplementary Figure 3B), ruling out this direct regulation of AMPK by AKT in vivo.

Mechanistically, modulation of mitochondrial respiration in skeletal muscle through inhibition of respiratory complex I increases intracellular AMP‐to‐ATP ratio and activates AMPK. Moreover, insulin/IGF‐1 induce mitochondrial respiration in myotubes in an AKT‐dependent manner^S1^. Previously, we reported that lack of AKT in skeletal muscle resulted in high AMP:ATP ratio and a significant reduction in complex I–dependent mitochondrial respiration (State 2 and State 3 respiration) in isolated mitochondria [17]. To test the hypothesis that insulin regulates AMPK activity via complex I–dependent mitochondrial respiration, we performed respiration assays on mitochondria isolated from the gastrocnemius muscles of M‐Control and M‐AKTDKO mice treated with insulin (2 U/kg body weight, intraperitoneally, 20 min). Loss of AKT led to a decrease in insulin‐dependent state 3, complex I respiration (Figure 5C), indicating that AKT signalling is required for insulin‐stimulated mitochondrial respiration.

Collectively, these data indicate a distinctive role of insulin in the regulation of AMPK, a catabolic kinase, potentially via modulation of complex I–dependent mitochondrial activity.

### 3.5 Inducible Deletion of AKT and AMPK in Adult Skeletal Muscle Are Sufficient to Cause Defect in Glucose Uptake in Skeletal Muscles and Alter Glucose Homeostasis

**FIGURE 6:**
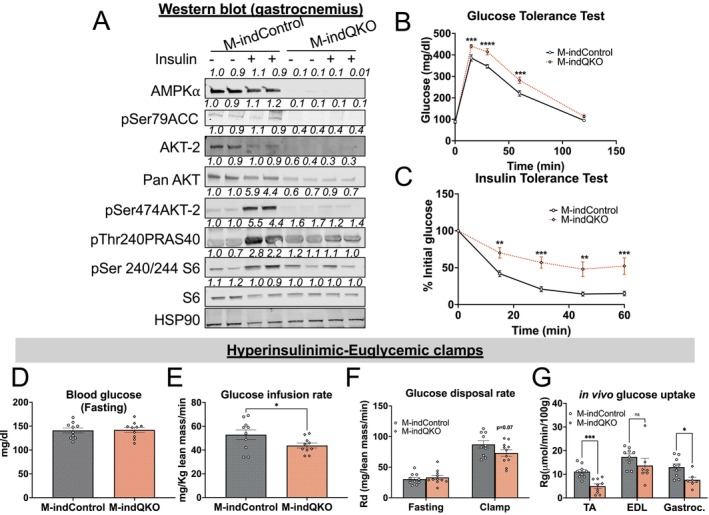
AKT and AMPK pathways in skeletal muscles are both required to regulate glucose uptake in adult skeletal muscles in response to insulin stimulation. (a) Western blot for AKT2, AMPK⍺, Pan AKT, S6, phosphorylation of AKT2, PRAS40, S6 and HSP90 in gastrocnemius muscle from control and experimental mice. Densitometric analysis of band intensities was performed, normalised to HSP90, and expressed as fold change relative to unstimulated control; values are shown above each Western blot (b) Intraperitoneal glucose tolerance test (2 g/kg) (*n* = 8 for control and 10 for M‐indQKO mice). (c) Insulin tolerance test (0.75 U/kg) (*n* = 8 for control and 10 for M‐indQKO mice). (d–g) Hyperinsulinemic‐euglycemic clamps were performed in control and M‐indQKO mice following 5 h fasting using a 2.5 mU/kg/min infusion of insulin. (d) Fasting blood glucose. (e) Glucose infusion rate normalised to lean mass. (f) Glucose disposal rate normalised to lean mass. (g) In vivo glucose uptake in skeletal muscle during hyper insulinemic‐euglycemic clamp (*n* = 7–9) (*p < 0.05, **p < 0.01, ***p < 0.001, ****p < 0.0001 vs. M‐indControl).

To specifically investigate the role of AKT and AMPK activity in glucose regulation in adult skeletal muscle, we utilised M‐indControl and M‐indQKO mice (lacking AKT1, AKT2, AMPKα1 and AMPKα2). Adult mice were injected with insulin and gastrocnemius muscles were harvested 20 min post‐injection for subsequent analysis by western blotting. Loss of AKT2 and AMPKα were confirmed using specific antibodies (Figure 6A). This was associated with a significant reduction in pAKT^Ser474^ signal (detected using an AKT2‐phospho specific antibody) and complete defect in the canonical insulin signalling pathway downstream of AKT such as pPRAS40^Thr240^ and pS6^Ser240/244^ in response to insulin (Figure 6A). In addition, AMPK signalling was effectively suppressed in M‐indQKO muscle, as demonstrated by a robust reduction in pACC^ser79^ in response to insulin (Figure 6A). In addition to defective insulin signalling, there was a ~15% reduction in muscle mass following 4–5 weeks post tamoxifen injections, without affecting body weight (Supplementary Figures 4A,B). Moreover, no significant difference in fasting and *ad libitum* plasma insulin levels was observed (Supplementary Figure 4C). However, M‐indQKO mice were significantly glucose and insulin intolerant (Figure 6B,C).

To assess insulin sensitivity directly, hyperinsulinemic‐euglycemic clamps in M‐indControl and M‐indQKO mice were performed. Consistent with the reduction in skeletal muscle weights, there was a significant reduction in lean mass in M‐indQKO mice (Supplementary Figure 4D). During the hyperinsulinemic portion of the clamp, M‐indQKO mice displayed a significant reduction in the glucose infusion rate and a trend towards decreased whole‐body insulin‐dependent glucose disposal rate (*p* = 0.07) when normalised to the lean mass (Figures 6E,F). No difference in glucose production rate was observed (Supplementary Figure 4E). Moreover, M‐indQKO mice exhibited a strong reduction in skeletal muscle glucose uptake, specifically in gastrocnemius and TA muscle (Figure 6G). The reduced glucose disposal in M‐indQKO mice was skeletal muscle‐specific as no defect in glucose uptake capacity in brown adipose tissue (BAT), heart and brain was observed (Supplementary Figure 4F). Notably, inducible deletion of muscle‐specific AMPKα alone (M‐indAMPKDKO) was insufficient to alter insulin sensitivity or affect glucose uptake capacity in skeletal muscle (Supplementary Figures 5A–C).

In summary, both AKT and AMPK signalling are required to regulate glucose uptake in skeletal muscle and systemic insulin sensitivity.

### 3.6 M‐indQKO Mice Display Impaired Insulin Dependent Glucose Uptake Capacity and GLUT4 Translocation

**FIGURE 7:**
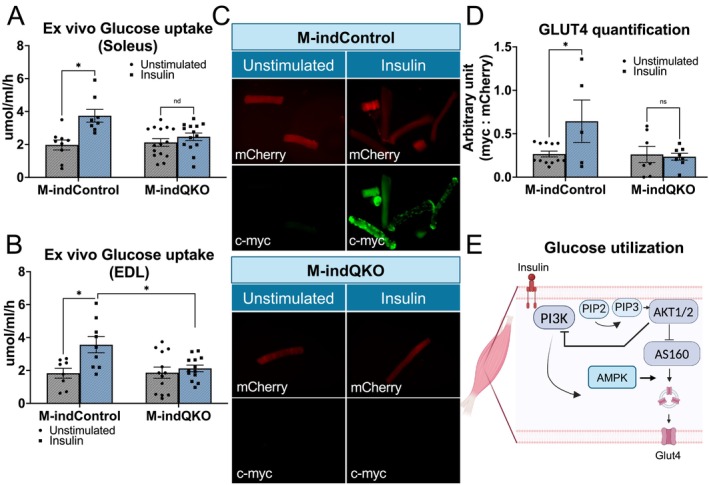
Both AKT and AMPK signalling are necessary to regulate GLUT4 translocation in skeletal muscles in response to insulin. (a,b) Ex vivo insulin‐stimulated glucose uptake was measured in isolated (a) soleus or (b) EDL from M‐indControl and M‐indQKO mice. (c) Representative confocal images of the abundance of plasma membrane GLUT4 relative to total GLUT4 in single murine muscle fibres isolated from flexor digitorum brevis (FDB) fibres‐expressing pLenti‐myc‐GLUT4‐mCherry. Transduced single muscle fibres from M‐indControl and M‐indQKO mice were incubated with or without insulin (10 nM) for 15 min. Non‐permeabilised FDB fibres following ± insulin treatment was stained with antibody against c‐myc to measure the abundance of GLUT4 expressed on the plasma membrane (top panel). In addition, red (mCherry) indicates total expression of the pLenti‐myc‐GLUT4‐mCherry lentivirus (middle panel). Images are representative of > 6 fibres from ≥ 3 different mice. (d) Quantified ratio of c‐myc/mCherry representing the GLUT4 translocated to the plasma membrane. (e) Predicted model of GLUT4 translocation and glucose uptake in response to insulin stimulation in skeletal muscle (*p < 0.05, **p < 0.01 vs. M‐indControl).

To further evaluate the cell autonomous effect of insulin on skeletal muscle glucose uptake, both EDL and soleus muscles were excised from M‐indQKO and M‐indControl mice and glucose uptake capacity was measured ex vivo. Consistent with the in vivo findings, fold change in glucose uptake capacity in insulin‐stimulated vs. unstimulated EDL and soleus muscle was completely blunted in M‐indQKO muscles as compared to M‐indControl muscles (Figure 7A,B). Notably, our laboratory previously reported that inducible deletion of AKT specifically in skeletal muscle (M‐indAKTDKO) does not have any effect on insulin‐stimulated glucose uptake capacity [17]. Next, we measured GLUT4 translocated to the plasma membrane in response to insulin stimulation in single muscle fibres from M‐indControl, M‐indAKTDKO and M‐indQKO FDB muscles. While both M‐indControl and M‐indAKTDKO fibres had enhanced GLUT4myc expression in insulin‐stimulated condition compared to the unstimulated fibres (Supplementary Figures 6A,B), no significant difference was observed in M‐indQKO muscle fibres (Figure 7C,D).

Altogether, these results suggest that insulin coordinates both AKT and AMPK signalling to regulate GLUT4 translocation and glucose uptake in skeletal muscle (Figure 7E).

## 4 Discussion

Decades of work have identified insulin‐dependent pathways that regulate GLUT4 trafficking, with AKT widely regarded as an obligate intermediate in this process. Much of this foundational knowledge stems from adipocyte models, where AKT2 associates with GLUT4 vesicles and phosphorylates the Rab‐GAP AS160 at Thr642 sites to promote GLUT4 translocation^S2^. Because adipocytes in culture are more responsive to insulin than muscle cells, they are often used as a preferred model to study insulin action. Moreover, owing to shared molecules in insulin signalling pathway and glucose transport processes, muscle and adipose cells are frequently employed interchangeably in research, with adipocytes often serving as surrogates for skeletal muscle. However, this methodological preference has inadvertently led to a lack of information about the cell‐autonomous contributions of AKT isoforms in skeletal muscle and adipose tissue in vivo, particularly in skeletal muscle. Given the distinct metabolic profiles and functions of these tissues, understanding how AKT regulates glucose transport in a tissue‐specific manner remains a critical and underexplored area of research. The present study directly addresses this gap and demonstrates, for the first time, intrinsic differences in insulin signalling networks between skeletal muscle and adipose tissue in vivo.

Our study, for the first time, demonstrates intriguing tissue‐specific disparities in the biology of AKT isoforms in muscle and adipocytes. Loss of AKT2 activity alone in adipose tissue leads to severe impairments in downstream signalling, including pAS160^Thr649^, glucose transport and systemic insulin resistance. This is consistent with longstanding in vitro studies and whole‐body AKT*2* knockout phenotype [3]. In contrast, skeletal muscle lacking AKT2 alone maintains intact AS160 phosphorylation and glucose transport, indicating substantial compensation by AKT1 in skeletal muscle but not in adipose tissue (Figure 1). Notably, AKT1 expression declines during adipocyte differentiation, which might limit its compensatory capacity in fat ^S3^. This likely reflects intrinsic differences in isoform expression, subcellular localisation, or binding to distinct scaffolding proteins ^S4^ of AKT in adipocytes contributing to the distinct biology of AKT1 and AKT2 in adipose and skeletal muscle.

In the present study, we focused on eWAT as a representative adipose depot due to the higher yield of primary adipocytes, facilitating ex vivo functional assays. While the canonical PI3K–AKT–AS160 signalling pathway regulating GLUT4 translocation is conserved in adipose tissue, depot‐specific differences exist, with inguinal white adipose tissue (iWAT) generally exhibiting greater insulin sensitivity and metabolic flexibility compared to eWAT^S5^. Therefore, although the fundamental signalling mechanisms are likely similar, the extent of AKT2‐dependent regulation of glucose uptake may differ between depots. Future studies will be required to determine whether these findings are conserved across subcutaneous adipose depots.

Despite these depot‐specific differences, the severe insulin intolerance observed in F‐AKT2KO is unlikely to be restricted to eWAT alone. Notably, DeFronzo et al. quantitatively demonstrated that skeletal muscle is the primary site for glucose disposal in humans. Following a meal, the majority of glucose is taken up by muscle and liver, with only 5% absorbed by adipose tissue. So how can the defects in glucose uptake capacity in eWATs of F‐AKT2KO mice account for systemic insulin resistance (Figure 2)? One possible explanation is that adipocytes regulate systemic metabolism primarily through lipolysis‐derived signals that influence liver and muscle insulin action. This is consistent with the studies demonstrating that adipocyte‐mediated lipolysis plays a central role in determining systemic insulin sensitivity ^S6–S8^.

Next, our study demonstrates that while the complete defect in pAS160^Thr649^ activity was observed in M‐AKTDKO muscle and F‐AKT2KO eWAT, the defect in glucose uptake capacity was observed only in F‐AKT2KO eWAT (Figure 1 and Figure 2). These results reveal the AKT‐AS160 axis is indispensable in adipose tissue but not strictly required for glucose uptake in skeletal muscle. This is consistent with other studies that implicate pAS160^Thr642^ is unlikely to be the sole AKT target that mediates GLUT4 translocation in skeletal muscle [16]. This divergence likely reflects tissue‐specific differences in the utilisation of Rab‐GAP proteins. While adipocytes rely predominantly on TBC1D4 (AS160), skeletal muscle expresses higher levels of TBC1D1, which is responsive to both insulin and AMPK signaling^S9–S11^. Consistent with this, we observed a robust increase in pTBC1D1^Ser231^ in M‐AKTDKO muscle, an AMPK‐regulated site associated with GLUT4 trafficking (Figure 5). Future studies will define the relative contribution of pTBC1D1^Ser231^ and pAS160^Thr642^ in controlling GLUT4 translocation and muscle glucose uptake. Together, these findings support a model in which AMPK‐dependent phosphorylation of TBC1D1 compensates for the loss of AKT signalling, enabling maintenance of glucose uptake in skeletal muscle. This highlights a fundamental divergence in GLUT4 regulatory mechanisms between muscle and adipose tissue and reinforces that these tissues are not mechanistically interchangeable despite sharing core components of the insulin signalling pathway.

Dysregulated or aberrant PI3K‐AKT activity underlies a variety of complex diseases including cancer and type 2 diabetes [7], making tight regulation of these signalling pathways essential. Like many signalling cascades, the PI3K‐AKT pathway is subjected to negative feedback regulation to assure that stimulatory signals are sensed and relayed in a transient manner. Consistent with this, insulin‐stimulated recruitment of AKT to the plasma membrane displays overshoot behaviour (a response where the initial maxima exceed the final steady state) and oscillations [34], hallmarks of dynamic negative feedback regulation. Although AKT is the most widely studied effector of PI3K signalling, and mediates many downstream metabolic effects, it is increasingly recognised that multiple PI3K‐dependent pathways operate in parallel to shape cellular responses^S12^. Interestingly, recent study in adipocytes identified the role of AKT in acutely restraining PI3K activity by limiting PIP3 production through post‐translational modification of the IRS, thereby limiting plasma membrane‐associated PI3K signalling [34]. Here, using genetic loss of function approach, we extend these findings and reveal a complex regulatory architecture of AKT signalling in skeletal muscle under both basal and insulin‐stimulated condition (Figure 3). Like adipocytes, loss of AKT leads to enhanced PI3K activity and PIP3 accumulation in skeletal muscle (Figure 4). Temporal analysis revealed that PIP3 accumulation in response to insulin is transient in M‐Control muscles, with detectable increase at early time points (10 min) but not at later stages (20 min). In contrast, M‐AKTDKO muscle exhibits sustained PIP3 accumulation at both early and later time points (Figure 4). These findings suggest that insulin driven PI3K activation is temporarily regulated, and that AKT plays a critical role in terminating this signal through negative feedback. Loss of AKT disrupts this feedback control, prolonging PI3K activity and permitting sustained activation of downstream PI3K‐dependent pathways. Collectively, these results underscore the dynamic and tightly regulated nature of PI3K–AKT signalling in skeletal muscle.

Several studies emphasise the reciprocal association between AKT and AMPK [25], the two important kinases in skeletal muscle that have redundant and counteracting functions in the regulation of metabolism and growth. As AKT signalling promotes glucose uptake and glycolysis, it stimulates ATP production and thereby indirectly prevents AMPK activation, a master cellular energy sensor that facilitates adaptation to ATP depletion. In contrast, prior exercise or AICAR stimulation enhances skeletal muscle insulin sensitivity ^S13^. These studies implicate intricate crosstalk between insulin‐mediated AKT and AMPK signalling. In addition, AKT has been previously reported to directly phosphorylate a carboxyl‐terminal residue on pAMPK^Ser487^ in vitro. However, our study demonstrates that this regulation is not apparent in skeletal muscle in vivo (Supplemental Figure 3). This might be attributed to the fact that tissues in vivo can respond differently due to factors like metabolic demands, cellular environment and interactions with other cell types.

Our studies demonstrate that activation of AMPK in AKT‐deficient muscle is not merely correlative but functionally required for insulin‐stimulated glucose uptake. Our previous ex vivo study using pharmacological inhibitors demonstrated that insulin regulates glucose uptake through a PI3K‐dependent mechanism in M‐AKTDKO muscle, and this effect was abolished when AMPK activity was inhibited. Here, our genetic epistasis experiments extend these findings by demonstrating that insulin coordinates both AKT and AMPK pathways to regulate glucose transport in skeletal muscle. These findings suggest that AMPK functions within a broader PI3K‐dependent signalling network. Together, these results support a model in which insulin signalling in skeletal muscle is distributed across multiple interconnected pathways downstream of PI3K, with coordinated input from AKT and AMPK playing a central role in regulating glucose uptake.

An unresolved question is how PI3K/PIP3 signalling contributes to AMPK activation in the absence of AKT. Our previous work reported elevations in the AMP:ATP ratio in M‐AKTDKO muscle under basal conditions, consistent with a metabolically stressed state associated with reduced complex I–dependent mitochondrial function. This impairment in oxidative metabolism may sensitise AMPK activation. Although we did not detect significant changes in AMP levels following insulin stimulation in M‐Control and M‐AKTDKO (potentially reflecting technical limitations in capturing rapid and transient nucleotide fluctuations in vivo), we consistently observed impaired complex I‐dependent mitochondrial respiration in M‐AKTDKO muscle, which was further exacerbated by insulin stimulation (Figure 5). These findings suggest that metabolic rewiring toward glycolysis, coupled with AMPK activation, contributes to the maintenance of glucose uptake in AKT‐deficient muscle. Additionally, our phospho‐proteomic analysis revealed significant alterations in pathways related to calcium signalling, including differential phosphorylation of CaMKKβ and ryanodine receptor 1 (RyR1). Given that AMPK can also be activated via CaMKKβ in response to increased cytosolic Ca^2+^, these findings provide a plausible alternative mechanism linking altered Ca^2+^ signalling and AMPK activation. We therefore propose that loss of AKT enhances PI3K signalling and rewires downstream pathways, leading to the activation of AMPK through either metabolic stress or calcium‐dependent mechanisms. Elucidating the precise mechanism linking PI3K/PIP3 to AMPK activation will be an important focus of future studies.

In summary, this work uncovers tissue‐specific roles of AKT isoforms, identifies AKT‐dependent negative feedback governing PI3K–PIP3 dynamics and reveals an unappreciated requirement for AMPK in insulin‐stimulated glucose uptake in AKT‐deficient muscle. These insights refine our understanding of insulin signalling architecture and may inform therapeutic strategies for metabolic disease, muscle disorders and cancer.

## Author Contributions

N.J. conceived the hypothesis, designed and performed experiments, analysed data and prepared the manuscript. L.L. designed and performed experiments and analysed data. M.G. and O.Y.O. provided technical assistance. W.H.D. designed the experiments, contributed to discussion and analysed the data. P.M.T. conceived the hypothesis, designed experiments, analysed data, and directed the project. All authors approved the final version of the manuscript.

## Funding

The authors have nothing to report.

## Ethics Statement

All animal experiments were reviewed and approved by the Institutional Animal Care and Use Committee (IACUC) at the University of Pennsylvania and were conducted in accordance with the ethical standards outlined in the 1964 Declaration of Helsinki and its later amendments, as well as all applicable US national regulations, including the NIH Guide for the Care and Use of Laboratory Animals.

## Conflicts of Interest

PMT's research contributing to this manuscript was conducted at the University of Pennsylvania while serving as a faculty member (2017–2025). PMT is currently an employee of Eli Lilly and Company; however, the research contributing to this manuscript, as well as the discussion and viewpoints expressed, are not affiliated with, nor endorsed by, Eli Lilly and Company. PMT is acting on their own in the preparation and submission of this manuscript.

## Supporting information


**Figure S1:** AKT signalling alone is not sufficient to regulate GLUT4 translocation in skeletal muscles in response to insulin. (A) Representative confocal images of FDB fibres‐expressing pLenti‐myc‐GLUT4‐mCherry lentivirus ± insulin (10 nM) for 15 min from M‐indControl and MindAKTDKO mice. Green‐myc represents the GLUT4 expression on plasma membrane in nonpermabilised FDB fibres following ± insulin treatment and stained with antibody against c‐myc (top panel). Red mCherry represents the FDB fibres expressing pLenti‐myc‐GLUT4‐mCherry lentivirus (middle panel). Images are representative of > 6 fibres from ≥ 3 different mice. (B) Quantified ratio of myc/mCherry representing the GLUT4 translocated to the plasma membrane (*p < 0.05 vs. M‐Control/M‐indControl).
**Figure S2:** Heatmap representation of all significant phosphosites. (A) Heatmap of the significantly regulated phosphosites in M‐Control gastrocnemius samples either unstimulated or stimulated with insulin (2 U/kg) for 20 min following an overnight fast. (B) Heat map of the significantly regulated phosphosites in M‐AKTDKO gastrocnemius samples either unstimulated or stimulated with insulin (2 U/kg) for 20 min following an overnight fast (cutoff: Log2(fold‐change) > 1, or p value < 0.05). Red indicated downregulated phosphosites and green indicated upregulated phosphosites.
**Figure S3:** AKT activation in response to insulin does not directly regulate AMPK at Ser485 in vivo. (A) Western blot for pSer485 AMPK and HSP90 in nutritional stressed myotubes ± MK2206 (10 μM for 6 h) followed by ± insulin (10 nM for 15 min). (B) Western blot for pSer485 AMPK and HSP90 in gastrocnemius muscle harvested from M‐Control and M‐AKTDKO mice treated with insulin (2 U/kg) for 20 min following an overnight fast.
**Figure S4:** Combined deletion of both AKT and AMPK cause the defect in muscle mass and glucose homeostasis in adult skeletal muscle: (A) Body weight of M‐indControl and M‐indQKO mice (n = 7–10). (B) Muscle mass from different muscle depot from M‐indControl and M‐indQKO mice (n = 8 for control and n = 12 for experimental mice). (C) Plasma insulin level in fasting and ad libitum state (n = 5–10). (D) Lean mass of M‐indControl and M‐indAKTDKO mice (n = 6–7). (E) Glucose production rate normalised to lean mass during hyperinsulinemic–euglycemic clamp (n = 9–10). (F) In vivo glucose uptake in extrahepatic tissue from M‐indControl and M‐indQKO mice (n = 9–10)) (*p < 0.05, ***p < 0.001 vs. M‐indControl).
**Figure S5:** Muscle‐specific AMPK signalling alone is not sufficient to regulate glucose uptake in skeletal muscle. (A) Intraperitoneal glucose tolerance test (2 g/kg) (n = 5–7). (B) Insulin tolerance test (0.75 U/kg) (n = 5–7). (C) Ex vivo insulin‐stimulated glucose uptake was measured in EDL and soleus muscles (n = 5–7) (*p < 0.05, **p < 0.01 vs. M‐indControl).
**Figure S6:** AKT signalling alone is not sufficient to regulate GLUT4 translocation in skeletal muscles in response to insulin. (A) Representative confocal images of FDB fibres‐expressing pLenti‐myc‐GLUT4‐mCherry lentivirus ± insulin (10 nM) for 15 min from M‐indControl and MindAKTDKO mice. Green‐myc represents the GLUT4 expression on plasma membrane in nonpermabilised FDB fibres following ± insulin treatment and stained with antibody against c‐myc (top panel). Red mCherry represents the FDB fibres expressing pLenti‐myc‐GLUT4‐mCherry lentivirus (middle panel). Images are representative of > 6 fibres from ≥ 3 different mice. (B) Quantified ratio of myc/mCherry representing the GLUT4 translocated to the plasma membrane (*p < 0.05 vs. MindControl).
