# Supplementary material for: Divergent AKT Signalling Mechanisms Regulate GLUT4 Translocation and Glucose Uptake in Skeletal Muscle and Adipose Tissue
Source: J Cachexia Sarcopenia Muscle. 2026 Jul 8;17(4):e70335. doi: 10.1002/jcsm.70335 (PMC13343733; doi:10.1002/jcsm.70335)
Supplement: Supplementary file 1 — Figure S1: AKT signalling alone is not sufficient to regulate GLUT4 translocation in skeletal muscles in response to insulin. (A) Representative confocal images of FDB fibres‐expressing pLenti‐myc‐GLUT4‐mCherry lentivirus ± insulin (10 nM) for 15 min from M‐indControl and MindAKTDKO mice. Green‐myc represents the GLUT4 expression on plasma membrane in nonpermabilised FDB fibres following ± insulin treatment and stained with antibody against c‐myc (top panel). Red mCherry represents the FDB fibres expressing pLenti‐myc‐GLUT4‐mCherry lentivirus (middle panel). Images are representative of > 6 fibres from ≥ 3 different mice. (B) Quantified ratio of myc/mCherry representing the GLUT4 translocated to the plasma membrane (*p < 0.05 vs. M‐Control/M‐indControl). Figure S2: Heatmap representation of all significant phosphosites. (A) Heatmap of the significantly regulated phosphosites in M‐Control gastrocnemius samples either unstimulated or stimulated with insulin (2 U/kg) for 20 min following an overnight fast. (B) Heat map of the significantly regulated phosphosites in M‐AKTDKO gastrocnemius samples either unstimulated or stimulated with insulin (2 U/kg) for 20 min following an overnight fast (cutoff: Log2(fold‐change) > 1, or p value < 0.05). Red indicated downregulated phosphosites and green indicated upregulated phosphosites. Figure S3: AKT activation in response to insulin does not directly regulate AMPK at Ser485 in vivo. (A) Western blot for pSer485 AMPK and HSP90 in nutritional stressed myotubes ± MK2206 (10 μM for 6 h) followed by ± insulin (10 nM for 15 min). (B) Western blot for pSer485 AMPK and HSP90 in gastrocnemius muscle harvested from M‐Control and M‐AKTDKO mice treated with insulin (2 U/kg) for 20 min following an overnight fast. Figure S4: Combined deletion of both AKT and AMPK cause the defect in muscle mass and glucose homeostasis in adult skeletal muscle: (A) Body weight of M‐indControl and M‐indQKO mice (n = 7–10). (B) Muscle mass from different muscle [file JCSM-17-e70335-s001.pdf]

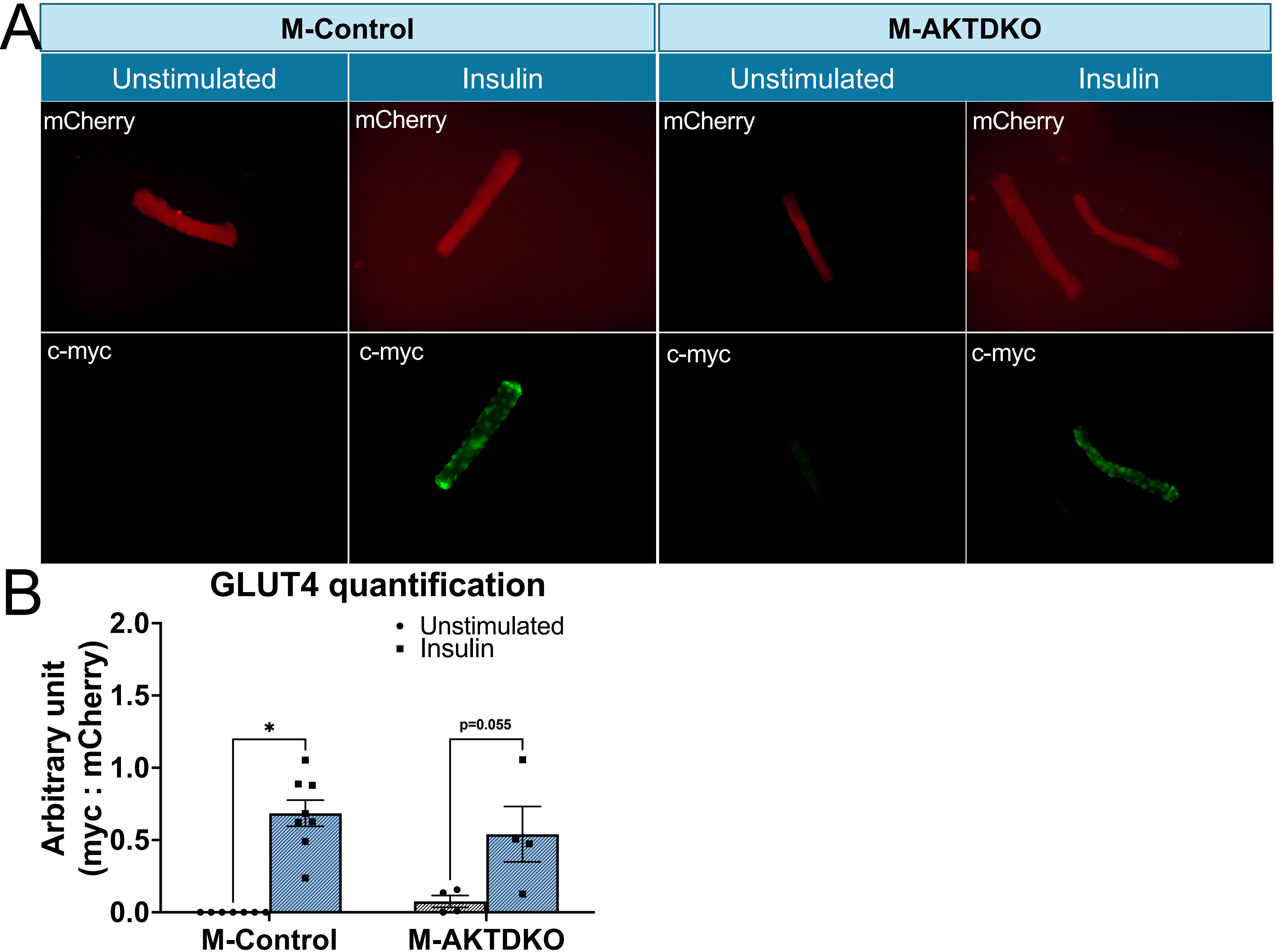
A
M-Control
M-AKTDKO
Unstimulated
Insulin
Unstimulated
Insulin
mCherry
c-myc
B
GLUT4 quantification
Arbitrary unit (myc : mCherry)
2.0
1.5
1.0
0.5
0.0
Unstimulated
Insulin
*
p=0.055
M-Control
M-AKTDKO

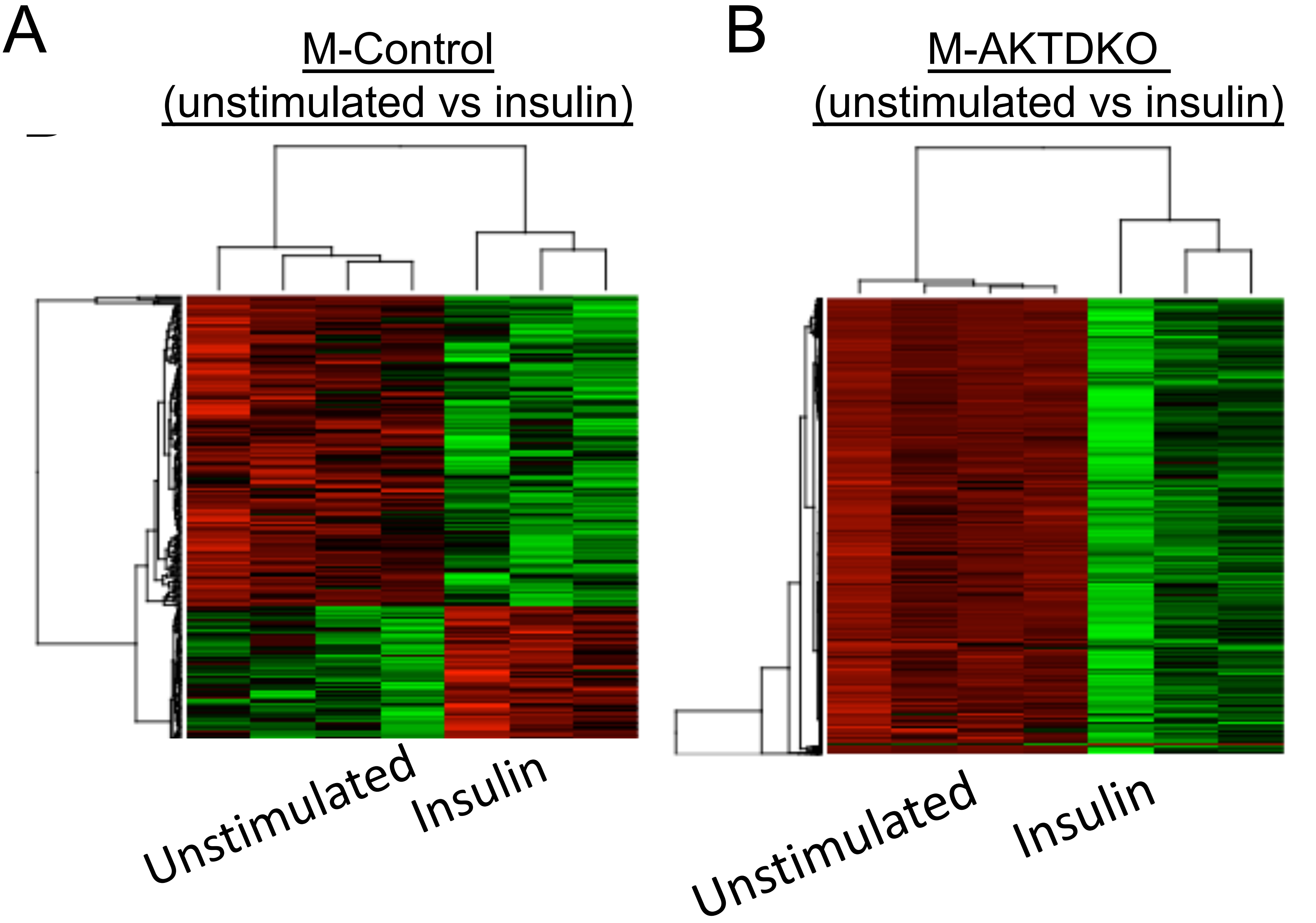
A
M-Control
(unstimulated vs insulin)
Unstimulated
Insulin
B
M-AKTDKO
(unstimulated vs insulin)
Unstimulated
Insulin

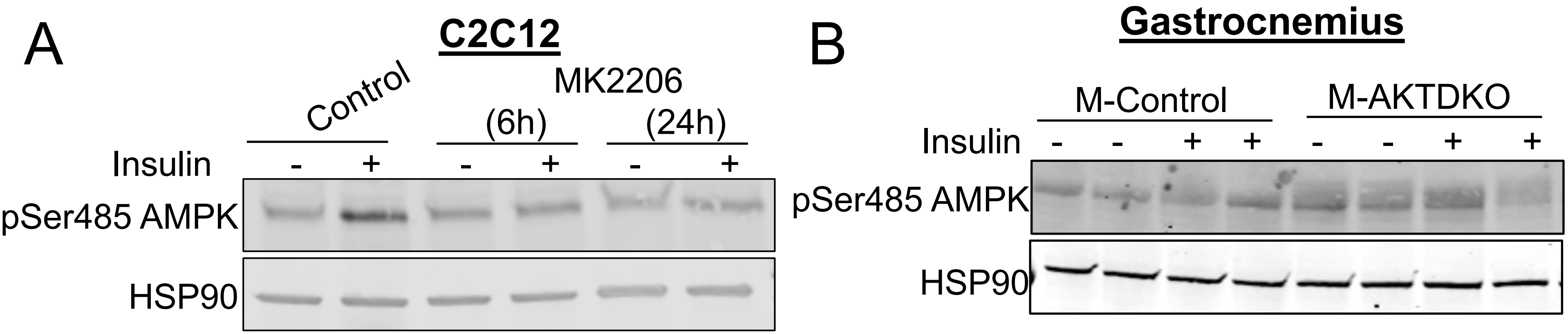

A
C2C12
Control
MK2206
(6h)
(24h)
Insulin - + - + - +
pSer485 AMPK
HSP90
B
Gastrocnemius
M-Control
M-AKTDKO
Insulin - - + + - - + +
pSer485 AMPK
HSP90

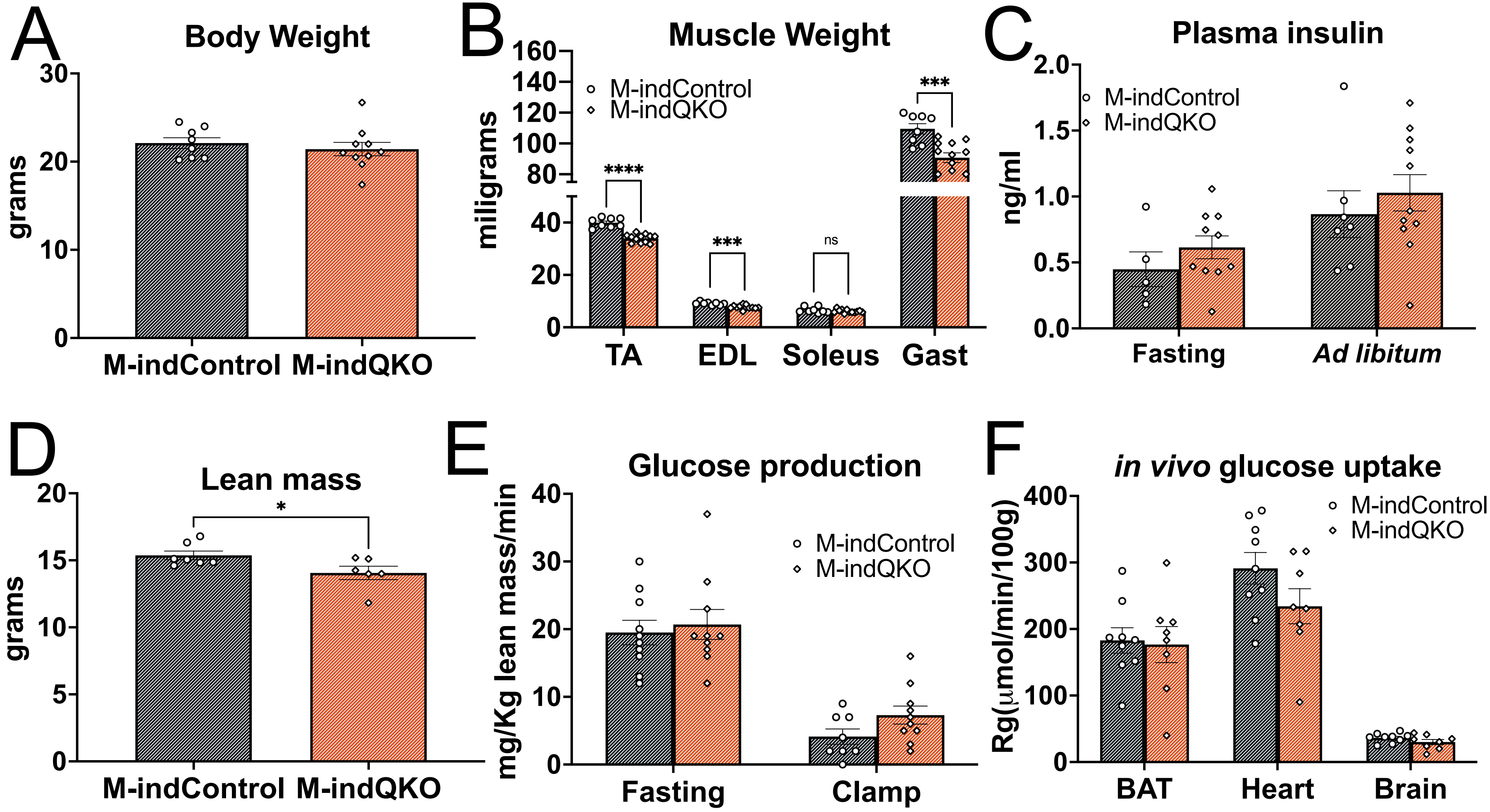
A
Body Weight
grams
30
20
10
0
M-indControl
M-indQKO
B
Muscle Weight
160
140
120
100
80
40
20
0
miligrams
M-indControl
M-indQKO
****
***
ns
***
TA
EDL
Soleus
Gast
C
Plasma insulin
2.0
1.5
1.0
0.5
0.0
ng/ml
M-indControl
M-indQKO
Fasting
Ad libitum
D
Lean mass
20
15
10
5
0
grams
*
M-indControl
M-indQKO
E
Glucose production
40
30
20
10
0
mg/Kg lean mass/min
M-indControl
M-indQKO
Fasting
Clamp
F
in vivo glucose uptake
400
300
200
100
0
Rg(μmol/min/100g)
M-indControl
M-indQKO
BAT
Heart
Brain

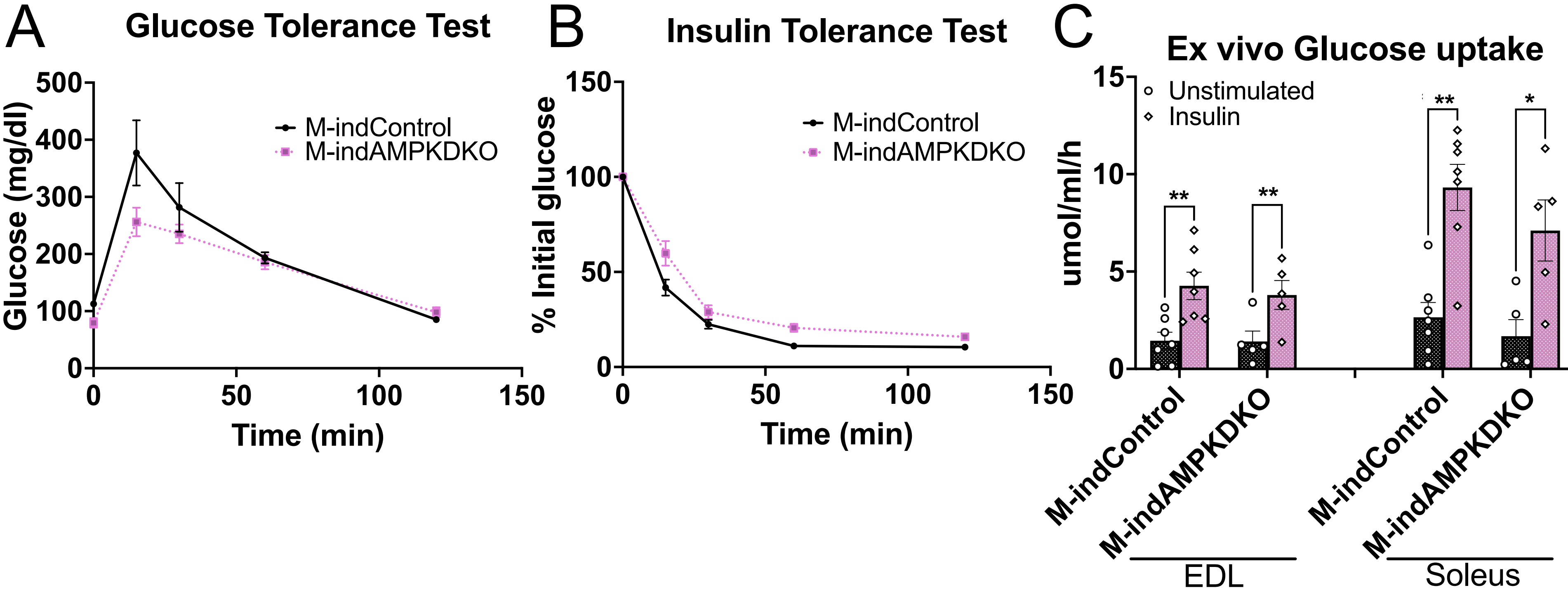

A
Glucose Tolerance Test
Glucose (mg/dl)
M-indControl
M-indAMPKDKO
Time (min)
B
Insulin Tolerance Test
% Initial glucose
M-indControl
M-indAMPKDKO
Time (min)
C
Ex vivo Glucose uptake
umol/ml/h
Unstimulated
Insulin
M-indControl
M-indAMPKDKO
M-indControl
M-indAMPKDKO
EDL
Soleus

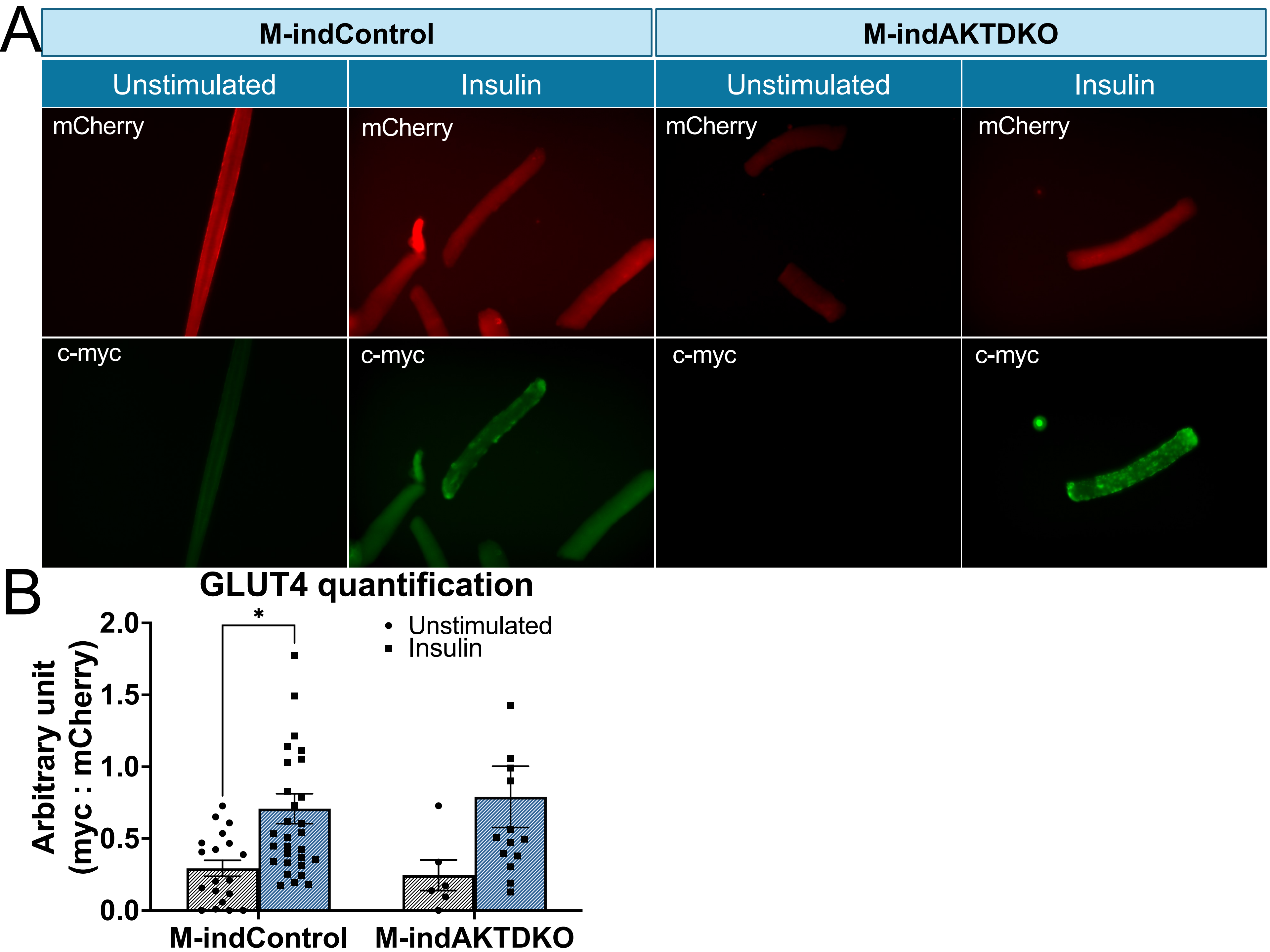
A
M-indControl
M-indAKTDKO
Unstimulated
Insulin
Unstimulated
Insulin
mCherry
mCherry
mCherry
mCherry
c-myc
c-myc
c-myc
c-myc
B
GLUT4 quantification
Arbitrary unit
(myc : mCherry)
2.0
1.5
1.0
0.5
0.0
*
Unstimulated
Insulin
M-indControl
M-indAKTDKO

Supplementary Figure legends

Figure S1: AKT signaling alone is not sufficient to regulate GLUT4 translocation in skeletal muscles in response to insulin. (A) Representative confocal images of FDB fibers-expressing pLenti-myc-GLUT4-mCherry lentivirus ± insulin (10 nM) for 15 minutes from M-indControl and M-indAKTDKO mice. Green-myc represents the GLUT4 expression on plasma membrane in non-permablized FDB fibers following ± insulin treatment and stained with antibody against c-myc (top panel). Red mCherry represents the FDB fibers expressing pLenti-myc-GLUT4-mCherry lentivirus (middle panel). Images are representative of >6 fibers from ≥3 different mice. (B) Quantified ratio of myc/mCherry representing the GLUT4 translocated to the plasma membrane. (*p<0.05 vs. M-Control/ M-indControl).

Figure S2: Heatmap representation of all significant phosphosites. (A) Heat map of the significantly regulated phosphosites in M-Control gastrocnemius samples either unstimulated or stimulated with insulin (2 U/kg) for 20 minutes following an overnight fast. (B) Heat map of the significantly regulated phosphosites in M-AKTDKO gastrocnemius samples either unstimulated or stimulated with insulin (2 U/kg) for 20 minutes following an overnight fast. (Cutoff: Log_2(fold-change) > 1, or p-value $\leq$ 0.05). Red indicated downregulated phosphosites and green indicated upregulated phosphosites.

Figure S3: AKT activation in response to insulin does not directly regulate AMPK at Ser485 *in vivo*. (A) Western blot for pSer485 AMPK and HSP90 in nutritional stressed myotubes ± MK2206 (10 uM for 6h) followed by ± insulin (10 nM for 15 minutes) (B) Western blot for pSer485 AMPK and HSP90 in gastrocnemius muscle harvested from M-Control and M-AKTDKO mice treated with insulin (2 U/kg) for 20 minutes following an overnight fast.

Figure S4: Combined deletion of both AKT and AMPK cause the defect in muscle mass and glucose homeostasis in adult skeletal muscle: (A) Body weight of M-indControl and M-indQKO mice (n = 7 -10). (B) Muscle mass from different muscle depot from M-indControl and M-indQKO mice (n-8 for control and n=12 for experimental mice). (C) Plasma insulin level in fasting and *ad libitum* state (n= 5-10). (D) Lean mass of M-indControl and M-indAKTDKO mice (n=6-7). (E) Glucose production rate normalized to lean mass during hyperinsulinemic-euglycemic clamp (n=9-10). (F) *In vivo* glucose uptake in extrahepatic tissue from M-indControl and M-indQKO mice (n=9-10).). (*p<0.05, ***p<0.001 vs. M-indControl).

Figure S5: Muscle-specific AMPK signaling alone is not sufficient to regulate glucose uptake in skeletal muscle. (A) Intraperitoneal glucose tolerance test (2 g/kg) (n = 5-7). (B) Insulin tolerance test (0.75 U/kg) (n=5-7). (C) *Ex vivo* insulin-stimulated glucose uptake was measured in EDL and soleus muscles (n = 5-7). (*p<0.05, **p<0.01 vs M-indControl).

Figure S6: AKT signaling alone is not sufficient to regulate GLUT4 translocation in skeletal muscles in response to insulin. (A) Representative confocal images of FDB fibers-expressing pLenti-myc-GLUT4-mCherry lentivirus ± insulin (10 nM) for 15 minutes from M-indControl and M-indAKTDKO mice. Green-myc represents the GLUT4 expression on plasma membrane in non-permablized FDB fibers following ± insulin treatment and stained with antibody against c-myc (top panel). Red mCherry represents the FDB fibers expressing pLenti-myc-GLUT4-mCherry lentivirus (middle panel). Images are representative of >6 fibers from ≥3 different mice. (B) Quantified ratio of myc/mCherry representing the GLUT4 translocated to the plasma membrane. (*p<0.05 vs. M-indControl).

Supplementary Methods

S2.1. Mice

All mice used in all experiments were on a C57/B6J background and were males. To generate muscle-specific M-AKT1KO, M-AKT2KO, M-AKTDKO, M-AMPKDKO and M-QKO knockout mice, floxed strains $Akt1_{loxP/loxP}$, $Akt2_{loxP/loxP}$, $Akt1_{loxP/loxP}$;$Akt2_{loxP/loxP}$, $AMPK\alpha1_{loxP/loxP}$, $AMPK\alpha1_{loxP/loxP}$;$AMPK\alpha2_{loxP/loxP}$ were crossed with mice carrying the Cre recombinase driven by a skeletal muscle actin promoter, ACTA1-Cre (Jackson Laboratory, stock number 006149). Littermate that lacked the HSA-Cre transgene served as controls. For skeletal muscle AKT deficiency in adult mice, floxed mice were crossed to mice containing the Cre recombinase-estrogen receptor fusion protein under the control of human ACTA-1 (HSA-ESR-CRE) (Jackson Laboratory, stock number 031934), allowing tamoxifen-inducible Cre-mediated recombination. AKT mice (8–12 weeks of age) for the indicated genotype that lack the HSA-ESR-CRE transgene (M-indControl) or mice positive for the transgene (M-indAKTDKO) were injected with tamoxifen IP (100 mg/kg) for 5 consecutives days to induce knockout. Experiments were performed 2 and 4 weeks later following the 5th day of tamoxifen.

Adipose-specific AKT2 knockout mice (F-AKT2KO) were generated by crossing Adipoq *Cre* positive male mice heterozygous for $Akt2_{loxp/loxp}$ were bred to females homozygous for $Akt2_{loxp/loxp}$ [S14] to generate Adipoq*Cre* positive experimental mice homozygous for $Akt2_{loxp/loxp}$ (F-AKT2KO). Adipoq*Cre* negative male mice homozygous for $Akt2_{loxp/loxp}$ served as controls. Mice between the ages of 10–14 weeks were used for the experiment.

All mice were housed at RT (22°C) unless specified otherwise. Animal use followed all standard guidelines of the Institutional Animal Care and Use Committee (IACUC) at the University of Pennsylvania and in accordance with NIH regulations.

S2.2. Tissue lysate and Western blotting

Muscles frozen in liquid nitrogen were powdered on dry ice, then transferred to cold RIPA (50mM Tris HCl, 1% Triton x100, 0.5% Sodium deoxycholate, 0.1% SDS, 150 mM NaCl, 2 mM EDTA) buffer supplemented with protease inhibitor cocktail tablets (Roche), and phosphatase inhibitor cocktail I and II (Sigma). Cell lysates were homogenized using tissue homogenizer (Fisherbrand™ 150), incubated for 10 min on ice and centrifuged at 13,600 g for 30 min at 4 °C. Alternatively, frozen epidydimal white adipose tissue samples (eWATs) were homogenized in RIPA buffer supplemented with protease inhibitor cocktail tablets, and phosphatase inhibitor cocktail II and III in a tissue lyser (QIAGEN). Cleared lysates were then used to determine total protein levels (BCA Protein Assay, Pierce). After dilution with sample buffer, equal protein amounts were loaded onto SDS gels and were separated on 4%–15% Mini-PROTEAN TGX pre-cast gels. The antibodies used for blotting are as follows: Rabbit monoclonal Cell Signaling: Phospho-AKT (Ser473/474) (#4060), AKT (pan) (#4691), Phospho-AKT2 (Ser474) (#8599S), pAMPK (Thr 172) (#2535), AMPK (#5831), AKT2 (#2964), Phospho-PRAS40 (Thr 246) (#2997S), S6 Ribosomal Protein (#2217), Phospho-S6 Ribosomal Protein (Ser240/244) (#5364), Phospho-AS160 (Thr642) (#8881), HSP90 (#4874); Phospho-PDPK1 (Ser241) (#3438), PDPK1(#5662), Myc-Tag (71D10) (#2278); donkey anti-rabbit (#926-32213) from LI-COR.

S2.3. Metabolic Measurement

For glucose tolerance test, mice were fasted overnight and injected with glucose (intraperitoneally, 2 g/kg). Blood glucose was monitored by tail bleeding at 0, 15, 30, 60 and 120 min after glucose injection. For insulin tolerance test, mice were fasted for 5 h then injected with insulin (intraperitoneally, 0.75 U/Kg body weight). Blood glucose levels were monitored by tail bleeding at 0, 15, 30, 45 and 60 min.

S2.4. Mitochondrial isolation

Gastrocnemius muscle harvested from M-Control and M-AKTDKO mice under unstimulated or insulin stimulated condition (2U/Kg body weight, intraperitoneally for 20 minutes) were immediately placed in H+ buffer (100mM KCl, 50mM Tris-HCL (pH 7.4), 5mM MgCl2, 1mM EDTA 1.8mM ATP; pH 7.2). Fat and connective tissues were removed, and muscle were minced in ice-cold H+buffer. Chopped tissue were transferred into tube containing protease medium (ratio ~ 2mL prot. / 0.5g tissue) and transferred quickly into 15mL conical tube containing fresh, ice-cold H-buffer+ after 2 minutes incubation on ice. Tissues were homogenized using motor-driven dounce homogenizer for 10 min and centrifuged at ~720g for 5 minutes. The supernatant collected was centrifuged at 10,000xg for 30 minutes @ 4°C. The palette was gently agitated to dislodge the loose, white flotsam from the pure, dark brown mitochondria. Mito pellet was resuspended in 4uL resuspension buffer (225mM Sucrose, 44mM KH2PO4, 12.5mM Mg-acetate, 6mM EDTA; pH 7.4) per milligram tissue weight. Total protein levels were determined using BCA protein assay (Pierce).

S2.5. Mitochondrial respiration

Isolated mitochondria (150 μg) were resuspended in MiR05 respiration medium (110 mM mannitol, 0.5 mM EGTA, 3 mM MgCl2, 20 mM taurine, 10 mM KH2PO4, 60 mM K lactobionate, 0.3 mM DTT, and 0.1% BSA [fatty acid free], adjusted to pH of 7.1 with KOH) and oxygen consumption measured using an Oroboros Oxygraph-2k high-resolution respirometer. Real-time mitochondrial oxygen consumption rates were collected at 37°C with constant stirring. To measure state 2 respiration, we added 10 mM Pyruvate and 5 mM Malate. For state 3 respiration, we added 2 mM ADP. To inhibit complex V after state 3 respiration, we added 1 μg/ml Oligomycin.

S2.6. Hyperinsulinemic-Euglycemic (Insulin) Clamp

Insulin clamps were performed in catheterized mice as described [S15]. using [³H]-glucose infusion to measure whole-body glucose turnover and tissue-specific uptake. Whole-body glycolytic rate was determined by the $3H_2O$ formation rate, and Skeletal muscle glucose storage was calculated as the difference between Rd and glycolysis [S16]. A 13-mCi intravenous bolus of 2-[^{14}C]-deoxyglucose ([^{14}C]2DG) was administered at 120 min to determine the Rg, an index of tissue-specific glucose uptake. Blood samples were collected at 122, 125, 135, 145, and 155 min to measure [^{14}C]2DG disappearance from plasma. At 155 min, mice were anesthetized and tissues immediately harvested, and freeze clamped. Plasma and tissue were processed are described earlier [S17].

S2.7. GLUT4 lentivirus preparation

Lenti-myc-GLUT4-mCherry lentivirus was packaged with a third-generation lentivirus system [S18]. HEK293T cells were seeded in a 6 cm dish at a density of 5×10^6 cells the day before transfection. 10 µg of the pLenti-myc-GLUT4-mCherry plasmid (Addgene, 64049) along with 5.2 µg pRC-CMV-Rev1b (Addgene, 164443), 1 µg pHDM-Tat1b (Addgene, 164442), 2 µg pHDM-VSV-G (Addgene, 164440), and 4 µg pHDM-Hgpm2 (Addgene, 164441) helper plasmids were co-transfected into HEK293T cells with the Lipofectamine™ 3000 Transfection Reagent (Invitrogen, L3000015). The media was changed after 12 h and then were collected after 48 h. Following centrifugation at 400 g for 4 min, the virus-containing supernatant was passed through a 0.45 µm filter. The viruses were further precipitated with PEG-it Virus Precipitation Solution (System Biosciences, LV810A-1) and dissolved in 200 µL DMEM.

S2.8*. Ex vivo* GLUT4 translocation assay

To assess the translocation of GLUT4 to the cell surface in muscle cells, single fibers were isolated from the flexor digitorum brevis (FDB) muscle of male C57BL/6N mice (Charles River Laboratories). These isolated fibers underwent transduction with myc-GLUT4-mCherry lentivirus

for a duration of 24 hours. Following transduction, the muscle fibers were exposed to either BSA-PBS or BSA with 10 nM insulin (Eli Lilly, Humulin R U-100) for another 15 minutes, after which the fibers were fixed using 4% paraformaldehyde for 10 minutes. Fibers were incubated overnight with Myc-Tag (71D10) rabbit monoclonal antibody (Cell Signaling Technology, #2278) at a dilution of 1:500. The following day involved three PBS washes, an hour-long treatment with anti-rabbit secondary antibody, Alex 488 (Invitrogen, #A11034) at a 1:1000 dilution, and another trio of PBS washes. Visualization was achieved through both brightfield and fluorescence microscopy using a Keyence BZ-X700 fluorescent light microscope with 20x objective. Fluorescence intensity was analyzed using the Fiji software package (version 2.15.0). The intensity of the anti-Myc signal, indicative of GLUT4 at the plasma membrane, was standardized against the mCherry signal, which reflects the overall presence of GLUT4.

S2.9. Sample preparation for phosphoproteomics

Gastrocnemius muscle harvested from overnight fasted M-Control and M-AKTDKO mice post BSA/insulin (intraperitoneally, 2U/Kg body weight for 20 minutes) injection were homogenized in 1 ml of cold 1% NP40 lysis buffer (50mM Tris-HCL, pH 8.0 with 1% NP40, phosphatase and protease inhibitors) using tissue homogenizer (Fisherbrand™ 150). Tissue lysates were incubated at 4°C for 15 minutes and centrifuged at maximum speed at 4°C for 10 minutes to separate the NP40 soluble fraction which is free from the contractile proteins. Protein concentration was determined using a BCA assay kit (Thermo Fisher Scientific). After cell lysis, proteomes were extracted using a buffer containing 200 mM EPPS pH 8.5, 8M urea, and protease/phosphatase inhibitors, as described earlier [S19]. Following lysis, 300 µg of each proteome was reduced with 5 mM TCEP. Cysteine residues were alkylated using 10 mM iodoacetimide for 20 minutes at RT in the dark. Excess iodoacetimide was quenched with 10 mM DTT. Each proteome was precipitated using methanol-chloroform. The proteomes were solubilized in 200 mM EPPS pH 8.5 containing Lys-C (Wako, 129-02541). Samples were digested

overnight at room temperature with vigorous shaking. The next morning trypsin was added to each sample and further incubated for 6 hours at 37º C. Acetonitrile was added to each sample to achieve a final concentration of ~33%. Each sample was labelled with ~750 µg of TMTPro reagents (ThermoFisher Scientific). Following confirmation of satisfactory labelling (>97%), excess TMT was quenched by addition of hydroxylamine to a final concentration of 0.3%. The full volume from each sample was pooled and acetonitrile was removed by vacuum centrifugation for 1 hour. The pooled sample was acidified, and peptides were de-salted using a Sep-Pak 50mg tC18 cartridge (Waters). Peptides were eluted in 70% acetonitrile, 1% formic acid and dried by vacuum centrifugation.

S2.10. Phosphopeptide enrichment and fractionation

A phosphopeptide enrichment was performed using a High-Select Fe-NTA Phosphopeptide Enrichment Kit (ThermoFisher Scientific). Following enrichment, the phosphopeptides were fractionated using an offline HPLC (basic pH reversed-phase separation). TMT-labeled phosphopeptides were solubilized in 5% acetonitrile/10 mM ammonium bicarbonate, pH 8.0 and separated by an Agilent 300 Extend C18 column (3.5 μm particles, 4.6 mm ID and 250 mm in length). An Agilent 1260 binary pump coupled with a photodiode array (PDA) detector (Thermo Scientific) was used to separate the peptides. A 45-minute linear gradient from 10% to 40% acetonitrile in 10 mM ammonium bicarbonate pH 8.0 (flow rate of 0.6 mL/min) separated the peptide mixtures into a total of 96 fractions (36 seconds). A total of 96 Fractions were consolidated into 12 samples and vacuum dried to completion. Each sample was desalted via stage tip and re-dissolved in 5% formic acid/ 5% acetonitrile for LC-MSMS analysis.

S2.11. Liquid chromatography separation and tandem mass spectrometry (LC-MS/MS)

Phosphorylation data were collected on an Orbitrap Eclipse mass spectrometer (ThermoFisher Scientific) coupled to a Proxeon EASY-nLC 1000 LC pump (ThermoFisher Scientific).

Fractionated peptides were separated using a 120 min gradient at 550 nL/min on a 35 cm column (i.d. 100 µm, Accucore, 2.6 µm, 150 Å) packed in-house. MS1 data were collected in the Orbitrap (120,000 resolution; maximum injection time – 50 ms; AGC 10 × 105). Charge states between 2 and 5 were required for MS2 analysis, and a 180 second dynamic exclusion window was used. MS2 scans were performed in the Orbitrap with HCD fragmentation (isolation window 0.5 Da; 50,000 resolutions; NCE 37.5%; maximum injection time 350 ms; AGC 1.375 × 105).

S2.12. Data analysis

A suite of in-house software tools were used to for .RAW file processing and controlling peptide and protein level false discovery rates, assembling proteins from peptides, and protein quantification from peptides as previously described [S20]. MS/MS spectra were searched using Sequest against a Uniprot Mouse database (downloaded in May 214) with both the forward and reverse sequences. Database search criteria are as follows: tryptic with two missed cleavages, a precursor mass tolerance of 50 ppm, fragment bin tolerance of 0.02, static alkylation of cysteine (+57.02146 Da), static TMT labeling of lysine residues and N-termini of peptides (+304.2071 Da), variable oxidation of methionine (+15.99491 Da) and variable phosphorylation on serine, threonine, and tyrosine (+79.966 Da). Phosphorylation site localization was determined using the AScore algorithm [S21] using a threshold of 13 corresponding to 95% confidence in site localization. TMT reporter ion intensities were measured using a 0.003 Da window around the theoretical m/z for each reporter ion. Proteins with <160 summed signal-to-noise across all channels and <0.5 precursor isolation specificity were excluded from the final dataset.

Supplementary References

S1. Das S, Morvan F, Jourde B, Meier V, Kahle P, Brebbia P *et al.* ATP citrate lyase improves mitochondrial function in skeletal muscle. *Cell Metab* 2015;**21**:868–876.

S2. Sano H, Kane S, Sano E, Mîinea CP, Asara JM, Lane WS *et al.* Insulin-stimulated phosphorylation of a Rab GTPase-activating protein regulates GLUT4 translocation. *Journal of Biological Chemistry* 2003;**278**:14599–14602.

S3. Hill MM, Clark SF, Tucker DF, Birnbaum MJ, James DE, Macaulay SL. A Role for Protein Kinase Bβ/Akt2 in Insulin-Stimulated GLUT4 Translocation in Adipocytes. *Mol Cell Biol* 1999;**19**:7771–7781.

S4. Liao W, Wang S, Han C, Zhang Y. 14-3-3 Proteins regulate glycogen synthase 3β phosphorylation and inhibit cardiomyocyte hypertrophy. *FEBS Journal* 2005;**272**:1845–1854.

S5. Wueest S, Schoenle EJ, Konrad D. Depot-specific differences in adipocyte insulin sensitivity in mice are diet- and function-dependent. Adipocyte 2012;1:153–156.

S6. Wang L, Pydi SP, Zhu L, Barella LF, Cui Y, Gavrilova O et al. Adipocyte Gi signaling is essential for maintaining whole-body glucose homeostasis and insulin sensitivity. Nat Commun 2020;11.

S7. Karpe F, Dickmann JR, Frayn KN. Fatty acids, obesity, and insulin resistance: Time for a reevaluation. Diabetes. 2011;60:2441–2449.

S8. Titchenell PM, Quinn WJ, Lu M, Chu Q, Lu W, Li C et al. Direct Hepatocyte Insulin Signaling Is Required for Lipogenesis but Is Dispensable for the Suppression of Glucose Production. Cell Metab 2016;23:1154–1166.

S9. Dokas J, Chadt A, Nolden T, Himmelbauer H, Zierath JR, Joost HG et al. Conventional knockout of Tbc1d1 in mice impairs insulin- and AICAR-stimulated glucose uptake in skeletal muscle. Endocrinology 2013;154:3502–3514.

S10. Kjøbsted R, Roll JLW, Jørgensen NO, Birk JB, Foretz M, Viollet B et al. AMPK and TBC1D1 regulate muscle glucose uptake after-but not during-exercise and contraction. 2019.

S11. Kjøbsted R, Hingst JR, Fentz J, Foretz M, Sanz MN, Pehmøller C et al. AMPK in skeletal muscle function and metabolism. FASEB Journal 2018;32:1741–1777.

S12. Vanhaesebroeck B, Guillermet-Guibert J, Graupera M, Bilanges B. The emerging mechanisms of isoform-specific PI3K signalling. Nat Rev Mol Cell Biol 2010;11:329–341.

S13. Kjøbsted R, Treebak JT, Fentz J, Lantier L, Viollet B, Birk JB et al. Prior AICAR stimulation increases insulin sensitivity in mouse skeletal muscle in an AMPK-dependent manner. Diabetes 2015;64:2042–2055.

S14. Leavens KF, Easton RM, Shulman GI, Previs SF, Birnbaum MJ. Akt2 Is Required for Hepatic Lipid Accumulation in Models of Insulin Resistance. *Cell Metab* 2009;10:405–418.

S15. Berglund ED, Li CY, Poffenberger G, Ayala JE, Fueger PT, Willis SE *et al.* Glucose metabolism in vivo in four commonly used inbred mouse strains. *Diabetes* 2008;57:1790–1799.

S16. Rossetti L, Lee Y, Ruiz J, Aldridge SC, Shamoon H, Boden G *et al.* Downloaded from journals.physiology.org/journal/ajpendo at Purdue Univ Lib TSS. 2024.

S16. Lantier L, Williams AS, Williams IM, Guerin A, Bracy DP, Goelzer M *et al.* Reciprocity between skeletal muscle ampk deletion and insulin action in diet-induced obese mice. *Diabetes* 2020;69:1636–1649.

S18. Pahl MC, Liu L, Pippin JA, Wagley Y, Boehm K, Hankenson KD *et al.* Variant to gene mapping for carpal tunnel syndrome risk loci implicates skeletal muscle regulatory elements. *EBioMedicine* 2024;101.

S19. Navarrete-Perea J, Yu Q, Gygi SP, Paulo JA. Streamlined Tandem Mass Tag (SL-TMT) Protocol: An Efficient Strategy for Quantitative (Phospho)proteome Profiling Using Tandem Mass Tag-Synchronous Precursor Selection-MS3. *J Proteome Res* 2018;17:2226–2236.

S20. Li J, Van Vranken JG, Pontano Vaites L, Schweppe DK, Huttlin EL, Etienne C *et al.* TMTpro reagents: a set of isobaric labeling mass tags enables simultaneous proteome-wide measurements across 16 samples. *Nat Methods* 2020 doi:10.1038/s41592-020-0781-4.

S21. Beausoleil SA, Villén J, Gerber SA, Rush J, Gygi SP. A probability-based approach for high-throughput protein phosphorylation analysis and site localization. *Nat Biotechnol* 2006;24:1285–1292.
